# Small extracellular vesicles isolation and separation: Current techniques, pending questions and clinical applications

**DOI:** 10.7150/thno.74305

**Published:** 2022-09-06

**Authors:** Yuanwang Jia, Li Yu, Tieliang Ma, Wenrong Xu, Hui Qian, Yaoxiang Sun, Hui Shi

**Affiliations:** 1 Jiangsu Key Laboratory of Medical Science and Laboratory Medicine, Institute of Stem Cells, School of Medicine, Jiangsu University, Zhenjiang, China.; 2 Department of Clinical Laboratory, The Affiliated Yixing Hospital of Jiangsu University, Yixing, China.; 3 Aoyang Institute of Cancer, Affiliated Aoyang Hospital of Jiangsu University, 279 Jingang Road, Suzhou, Jiangsu, China.

**Keywords:** Small extracellular vesicles, exosomes, isolation techniques, clinical applications

## Abstract

Extracellular vesicles, especially small extracellular vesicles (sEVs) are now accepted as important messengers in cell-to-cell communication and as a promising drug delivery platform. They are involved in nearly all physiological and pathological processes and are involved in disease diagnosis and therapy. However, their heterogeneity of physicochemical properties and functions is not fully understood, which hinders further clinical applications. To obtain highly bioactive sEVs with both high yield and purity, will certainly facilitate their future study and application. This review informs up-to-date research on frequently-used and cutting-edge technologies of sEVs isolation and makes a deep comparison and analysis of different methods, including their advantages, limitations and applications. Pending questions about the inherent property of these small vesicles as well as isolation strategies are discussed. Additionally, an overview of their applications in disease diagnosis and treatment, including some of the on-going clinical trials, are also reviewed.

## Introduction

**Figure 1:**
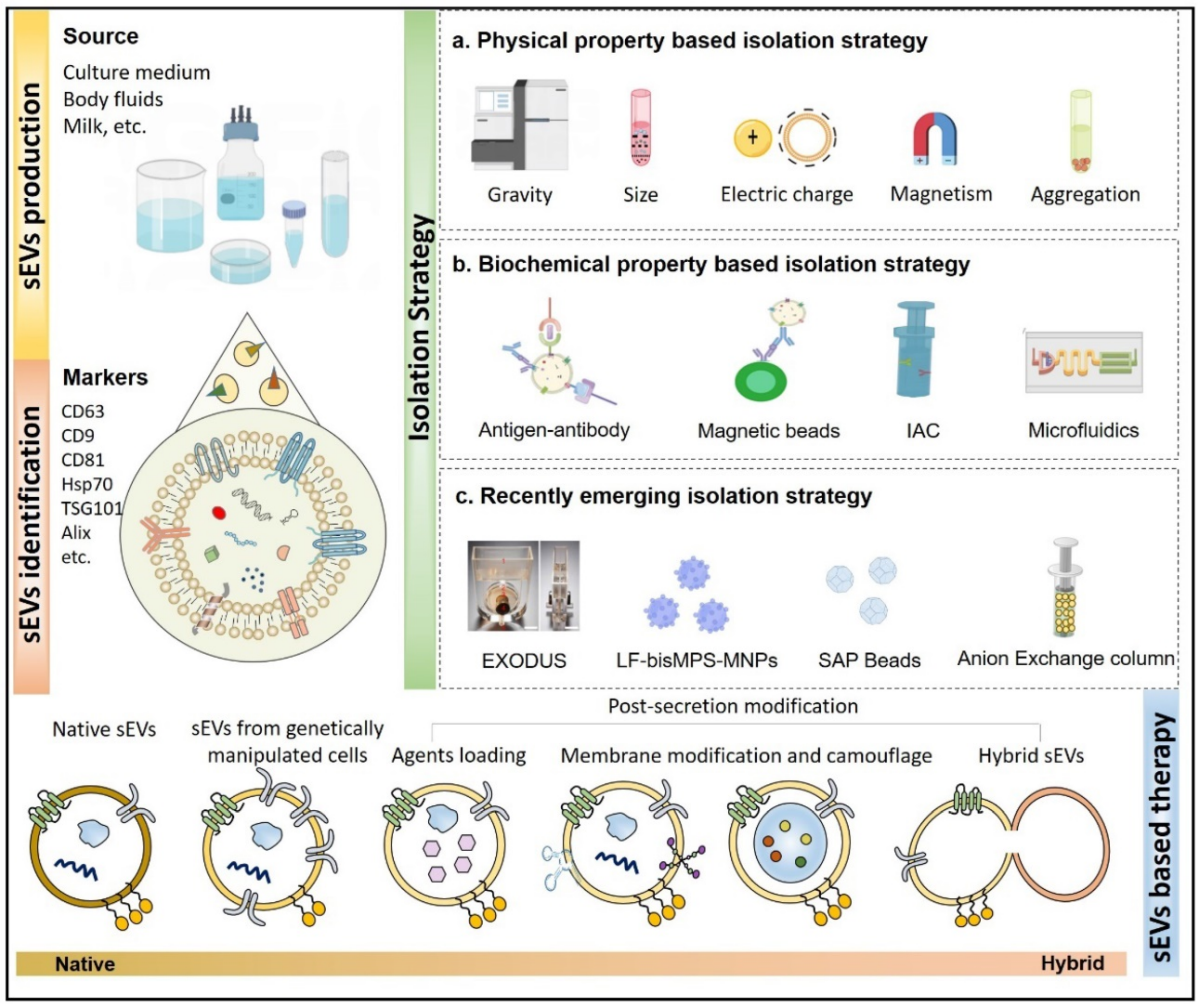
** Isolation and modification of sEVs.** sEVs can be isolated from cell or tissue culture medium, body fluids such as blood, urine, hydrothorax, ascites, milk, even beer and juice from plants. The isolated sEVs, particularly exosomes are usually found to express markers like CD63, CD81, CD9, Hsp70, TSG101, Alix and negatively express proteins such as calnexin. The existing methods developed to separate these vesicles are generally based on their physical or biochemical properties. Natural sEVs as well as tailored vesicles can bring great potential in disease treatment.

Extracellular vesicles (EVs) are a heterogeneous group of membrane-structured vesicles that are actively released by almost all types of cells and are found in various human body fluids such as blood, urine, saliva and ascites. Small extracellular vesicles (sEVs) usually refers to EVs smaller than 200 nm in diameter, and are the representative EV types most widely studies for their roles in different physiological and pathological conditions. They are broadly reported to transfer bioactive components (nucleic acids and proteins) from donor to recipient cells, thus mediating information exchange between cells 1. A growing number of studies have shown that sEVs play an important part in occurrence, diagnosis, and treatment of diseases and also as a new nano-platform for drug delivery (**Figure 1**). However, there are several challenges that still exist for the clinical applications of sEVs. For example, there is currently no standardization in the techniques for storage 2, dosage, and administration of sEVs 3. More importantly, the heterogeneity in the physicochemical properties and functions of sEVs is not fully understood. Additionally, the biological fluids where the sEVs circulate also contains various particles with properties overlapping those of the sEVs. Therefore, particular isolation techniques are of great importance since they are largely related to the physicochemical properties and contents of sEVs 4. Although EV separation methods are constantly updated, most of the currently available isolation techniques usually do not guarantee the purity and yield of the EVs at the same time; meanwhile, destruction of vesicle integrity is a risk that may hinder the accuracy of subsequent experiments. Some isolation techniques are multi-step and time-consuming, with low repeatability, which makes them unable to meet the actual clinical requirement 5, 6. In this review, we will focus on the existing isolation technologies in detail, discussing frequently-used as well as novel methods, and analyze their advantages and disadvantages. In addition, the challenges and future directions for research and clinical applications are also discussed. This review is aimed to provide the audience, whether an experienced researcher or a new hand in the field of EVs, with a full-scale understanding of sEVs isolation strategies to further facilitate their study and the future translational applications from bench to bedside.

## EV Characteristics, Biogenesis, and Cargos

EVs are lipid bilayer-encapsulated nanoparticles with a size of 50-1000 nm 7 that are present in different biological fluids 8, such as blood 9, urine 6, cerebrospinal fluid 10 and others 11. The obtained EVs may contain proteins, nucleic acids, lipids and metabolites 12, which might be similar or different from that of their cells of origin. The components can vary depending on distinct regulatory sorting mechanisms of their producing cells 13. EVs can transfer these contents from donor to recipient cells, mediating information exchange between cells 14, 15. EVs are shown to be highly heterogeneous in both structures and biological functions 16. The classification criteria for the subtypes of EVs have not yet been unified. According to MISEV2018 17, EVs can be divided into medium/large EVs (>200 nm) and small EVs (<200 nm) based on their physical properties of EVs. EVs can also be classified into apoptotic bodies (50-1000 nm in diameter), microvesicles (MVs) (100-1000 nm), and exosomes (40-160 nm, average~ 100 nm) based on their origin. Another way to classify EVs is according to their biological composition such as the presence of the surface protein CD63 18. Additionally, prevailing conditions are used to distinguish EVs as large oncosomes, hypoxic EVs, and podocyte EVs. Beyond that, as of now, there are still many EV particles whose functions and contents are undiscovered, and therefore, need to be characterized.

**Figure 2:**
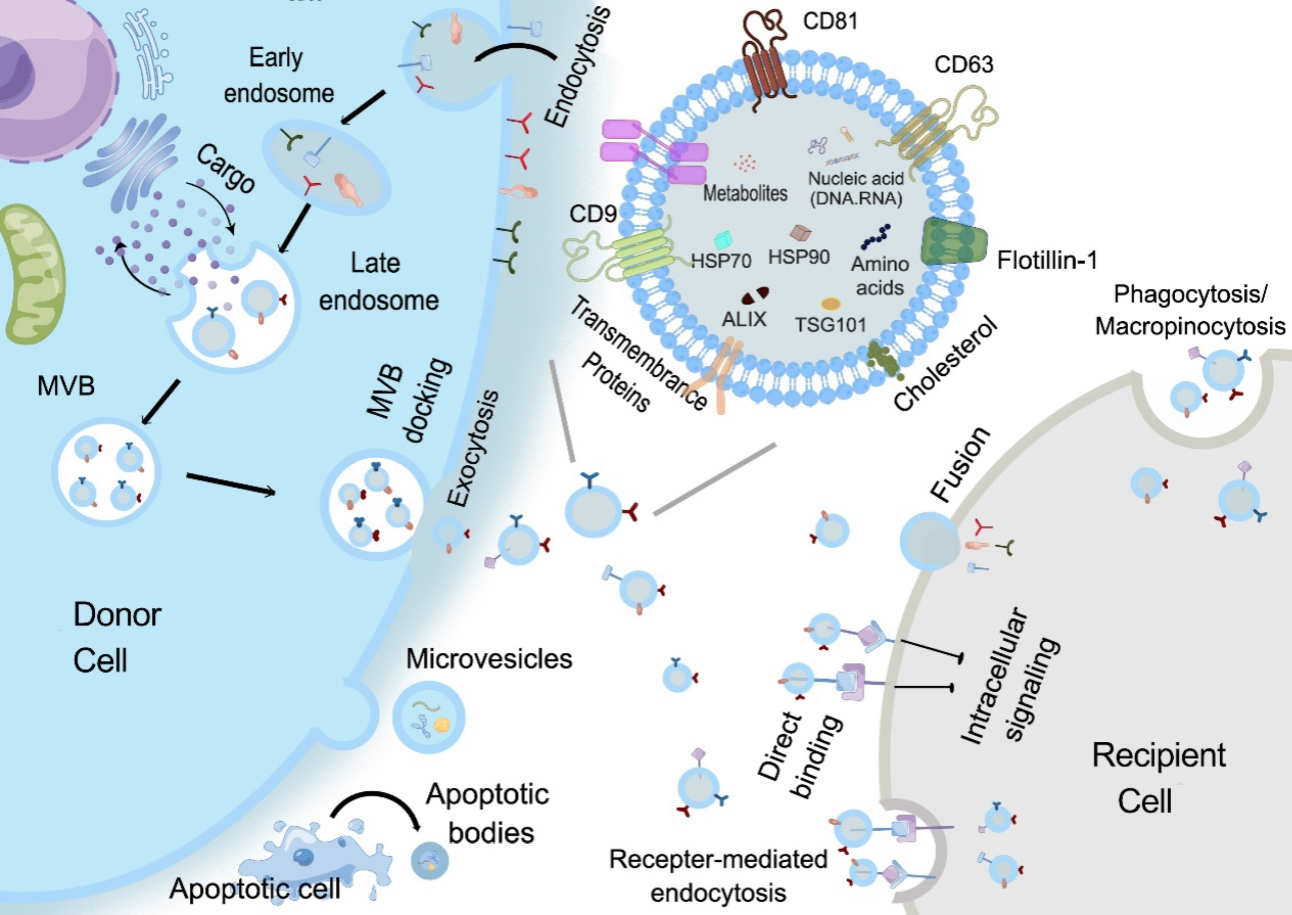
** Biogenesis and Cellular uptake of EVs.** Apoptotic bodies are formed by membrane folding, invagination and shedding with organelles and nuclear debris. MVs are formed by directly outward budding of plasma membranes. As for exosomes, firstly, the invagination of the plasma membrane forms a cup-shaped structure that includes cell surface proteins and some components such as proteins, lipids, and metabolites in the extracellular environment, that is, early sorting endosomes (ESE). ESE then develops into late sorting endosomes (LSEs), which invaginate to form intraluminal vesicles (ILVs), while components in the cytoplasm also enter the ILVs, and then LSEs form multivesicular bodies (MVBs). Finally, the MVBs fuse with the plasma membrane and release exosomes. The released exosomes taken up by recipient cells mainly through three ways: (1) Exosomes bind to cell membrane surface receptors. (2) Exosomes fuse directly with the cell membrane to release the contents. (3) Exosomes directly enter the cytoplasm in a complete form through cell pinocytosis or phagocytosis 19. The complex biogenesis, selection and transfer mechanism are responsible for the high heterogeneity of sEVs, which brings uncertainty and challenges to the standardization of isolation methods.

With respect to the biogenesis of EVs, apoptotic bodies are released by dying cells, which are seldomly used for study possibly due to their large and uneven particle size. MVs are formed by the direct outward budding of plasma membranes 18. Presently, most studies are focused on the potential of sEVs, especially exosomes in regenerative medicine. The specific process of exosomes biogenesis is recognized as a “swallow and spit” process. At the very beginning, the invagination of the plasma membrane forms a cup-shaped structure termed early sorting endosome (ESE) containing cell surface proteins and other biological substances (e.g., proteins, lipids, and metabolites). ESE then develops into late sorting endosomes (LSEs), which invaginates to form intraluminal vesicles (ILVs), packaging cytoplasmic contents. LSEs then form multivesicular bodies (MVBs) that finally fuse with the plasma membrane and release the exosomes 19. Compared with other types of EVs, exosomes are smaller in size and have specific markers such as CD9, CD63, CD81, HSP70, HSP90, Flotillin 1 and TSG101 20, 21 (**Figure 2**). Given that latest guidelines suggest the use of “EVs” to generally denote a heterogeneous extracellular vesicle population, and “exosomes” are defined as small extracellular vesicles that are released upon the exocytosis of MVBs filled with ILVs, in this review, the general term “small extracellular vesicles (sEVs)” in this review will be used as defined in the latest MISEV guideline.

Structurally, the sEVs phospholipid membrane bilayer provides a natural protection for their cargos and makes them highly biocompatible and favorable for cell-cell communication 22. The exposed phosphatidylserine regulates various pathophysiological processes, including inflammation, immune responses, coagulation, and neuronal regeneration 23, while the glycoconjugates (including proteoglycans and glycoproteins) participate in cell growth, migration, differentiation, tumor invasion, host-pathogen interactions, and transmembrane signaling 24. Moreover, some membrane proteins can also be inherited from their parent cells thus maintaining certain targeting properties. The specific cargos transported by the sEVs can include proteins, nucleic acids and metabolites, which could reflect the status of their parental cells 25. Although the exact mechanisms associated with distinct cargo sorting in sEVs are still unclear, several possible ways for contents loading have been discovered. Proteins can be sorted into MVBs by the regulation of tetraspanin-enriched microdomains or in an ubiquitin-dependent manner with the assistance of endosomal sorting complex required for transport (ESCRT). RNAs are shown to gain entrance with the help of factors such as Ago2, hnRNPA2B1, HuRand adenylation at the 3' end of miRNAs. In short, the protective and partial targeting abilities of sEVs, as well as their dynamic and specific cargo, bestow on them the potential to be ideal candidates for disease diagnosis and therapy 25-28.

## EV separation techniques

Given their multiple functions and clinical translation potential, to obtain sEVs with high yield and quality is of great significance. Currently, many techniques have been developed for sEVs separation which largely dependent on their biophysical and/or biochemical traits, such as the size, density, shape as well as specific surface markers. Both the inspection or research requirements and the complexity of the biological fluids where sEVs circulating should be taken into careful consideration when one particular method is chosen for the vesicle isolation. As for the complexity of samples, many non sEVs interferences such as lipoprotein in plasma, uromodulin (Tamm-Horsfall protein) in urine and surfactants bronchoalveolar lavage fluid 17, show the potential to co-isolate with sEVs to influence the subsequent observation. Specific clinical or research demands should also be considered. When using size exclusion chromatography (SEC) method, the product may be contaminated by abundant serum proteins but the yield is high, which makes SEC a suitable way for research that requires more on quantity, such as RNA analysis 29. Plasma samples used for liquid biopsy requires small sample volume with high yield 30. A batch of commercial kits based on precipitation thus become good options. Moreover, the selection of isolation technique also affects the structural integrity and functional activity of sEVs 31. For example, sEVs isolated by different methods (e.g. ultracentrifugation and SEC) show discrepant function in endothelial cell migration 31. Therefore, to choose the most appropriate method to isolate sEVs and even the subpopulations can allow better understanding of the vesicle biology and function before their clinical translation. Ideal isolation strategy with high-purity, high-yield, structural and functional integrality is still urgently needed 22, 32. The most frequently-used and cutting-edge sEVs isolation techniques will be discussed in detail in the following sections (**Table 1**).

### Ultracentrifugation (UC)

#### Differential Ultracentrifugation

**Figure 3:**
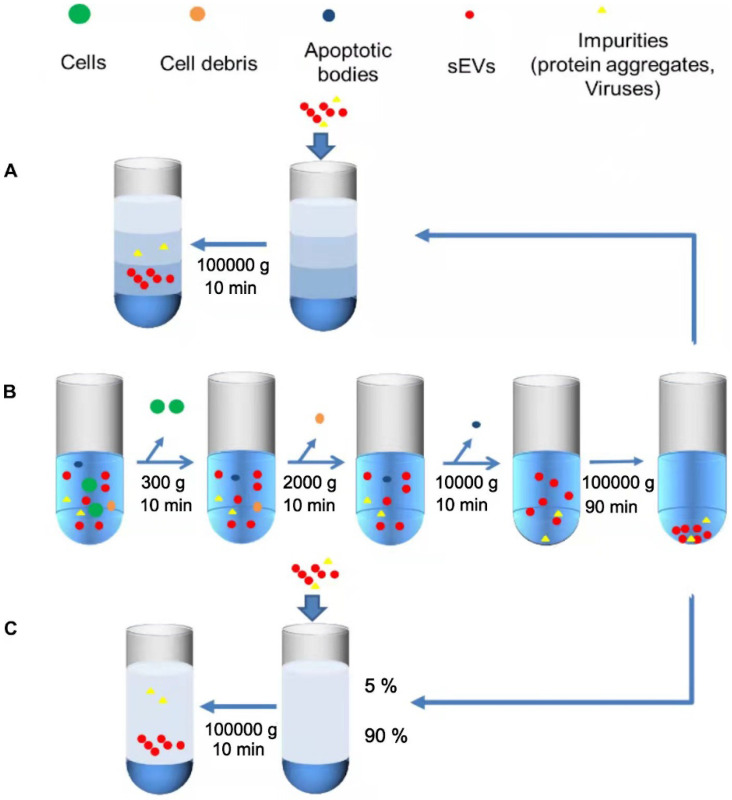
** Simplified illustration of ultracentrifugation of sEVs.** A. Isopycnic density gradient centrifugation. First, the samples are centrifuged at 300 ×*g*, 2000 ×*g* and 10,000 ×*g* to remove larger cells, cell debris and dead cells. Secondly, the sEVs are isolated by ultracentrifugation twice at a speed of more than 100,000 ×*g*. B. Differential Ultracentrifugation. Impurities are firstly removed by low-speed centrifugation (such as UC), and then the separated samples are added to the constructed density medium (3%, 35%, 45%, 90%) for separation. C. Rate zone ultracentrifugation. Construct high-density media (90%) at the bottom of the test tube as a buffer, operating steps are similar to DGC.

Differential ultracentrifugation is the most commonly used “gold standard” technique for sEVs isolation 30, which involves the fractionation and separation of substances with different densities and sizes by using different centrifugal speeds and forces (**Figure 3B**). The first few simple steps are performed to remove dead cells, cell debris, and large extracellular vesicles 22. The pellet thus obtained is resuspended in PBS, and a final ultracentrifugation step is performed to eliminate contaminating proteins. Centrifugation speed is selected according to the experimental requirements, and the temperature is maintained at 4 °C throughout the process to ensure that protease, DNase, and RNase is inactive 33. Finally, the characterization analysis of sEVs can be performed 34. This technique is simple to operate 35, low-cost, does not require extensive expertise or additional materials, which makes it reproducible and suitable for large-volume samples. Ye et al. has performed a characterization experiment using flow cytometry and showed that when sEVs were separated from plasma, UC had the highest isolation purity compared to other size- based or precipitation methods. Therefore, UC can be preferentially selected when separating sEVs from plasma 36. However, the results obtained by ultracentrifugation are relatively difficult to control, and easy to be affected by the type of biological material, the specific type of rotor, and the centrifugation time 37. In particular, when these features are not properly documented, the comparison between studies will not be convincing 38. Other limitations exist such as long time-consumption 39 and large output variation, which may also be affected by different operations 40. Under the external force of high-speed rotation, the structural and biological integrity of sEVs could be impaired 41. The quantitative and qualitative variation of samples could affect the accuracy of subsequent observations 42. The low particle recovery rate also makes this method unsuitable for small sample separations 43, 44. More importantly, while differential ultracentrifugation is now recognized as a high-purity isolation strategy, there is a possibility of co-segregation of contaminants, such as residual soluble protein 31, 40 and enriched particles with indistinguishable density or size, including microvesicles, non-vesicles, protein aggregates, and lipoproteins 45, 46. This method has been further improved by using isopycnic gradient ultracentrifugation. Another study described a new optimization method based on diluting serum with PBS to reduce viscosity, and prolonging the first UC cycle, followed by four more UC cycles. This approach was experimentally shown to remove 95% of serum proteins with no significant loss of sEVs when compared with size exclusion chromatography (SEC), along with providing sEVs of high purity 29.

#### Isopycnic density gradient centrifugation

Isopycnic density gradient centrifugation, an improvement on differential ultracentrifugation, is a density-based isolation technique that uses a density gradient tube and is based on the principle that objects with a specific density will remain suspended in a liquid layer with similar density after centrifugation 47 (**Figure 3A**). Isodensity centrifugation is a good solution for the problem of co-precipitation that is caused by overlapping physical properties when using UC for sEVs isolation. By constructing a density gradient medium, such as a sucrose medium, which gradually increases from top to the bottom of the centrifuge tube, sEVs settle along with the corresponding isodensity area under centrifugal force, and most contaminants are thus removed 22. Although both the purity and isolation efficiency are improved when compared with UC 48, isodensity centrifugation still possesses several disadvantages, such as the relatively complicated operation and expensive equipment 37. Suchi Gupta and colleagues have proposed a one-step sucrose cushion-buffered centrifugation (SUC) method, where body fluid or cell supernatant are added directly onto the sucrose cushion, thereby removing the previous preconcentration step, and then the sucrose cushion is collected and centrifuged to obtain sEVs after dilution with PBS. This method can improve the yield and integrity of sEVs produced 49. Kang Li et al. improved the existing method by proposing cushioned-density gradient ultracentrifugation (C-DGUC), where iodixanol buffer is used as a density gradient medium for the concentration of sEVs, which are further isolated by density ultracentrifugation. The iodixanol buffer can better maintain the physical and biological integrity of sEVs better. Moreover, as iodixanol is biologically inert and compatible, it need not be removed, eliminating an additional step. C-DGUC greatly improves sEVs yield and purity, and it has been demonstrated that sEVs can be extracted from plasma and urine, which is promising for clinical research and diagnosis 42.

#### Rate zone ultracentrifugation

Compared with isodensity ultracentrifugation, rate zone ultracentrifugation (RZC) is mainly based on particle diameter and can be used to separate particles with the same density but different diameters (**Figure 3C**) 50. For example, RZC can separate platelets from EV fractions 51. RZC has previously been used to isolate viruses, DNA 52, and other nanoparticles 51. The density of the medium in the RZC centrifuge tube should be a linear gradient from top to bottom of the tube, which is lower than that of the experimental sample. In addition, the linear gradient must be more viscous than the samples to prevent any mixing with the gradient when loading the sample, thus ensuring that the distance and speed of the particles moving in the centrifuge tube are mostly dependent on the particle diameter 53. However, all the particles will settle to the bottom of the tube as long as the time is sufficient; therefore, it is necessary to control the time strictly and place a high-density medium at the bottom of the tube as a buffer zone 46.

### Size Exclusion Chromatography

**Figure 4:**
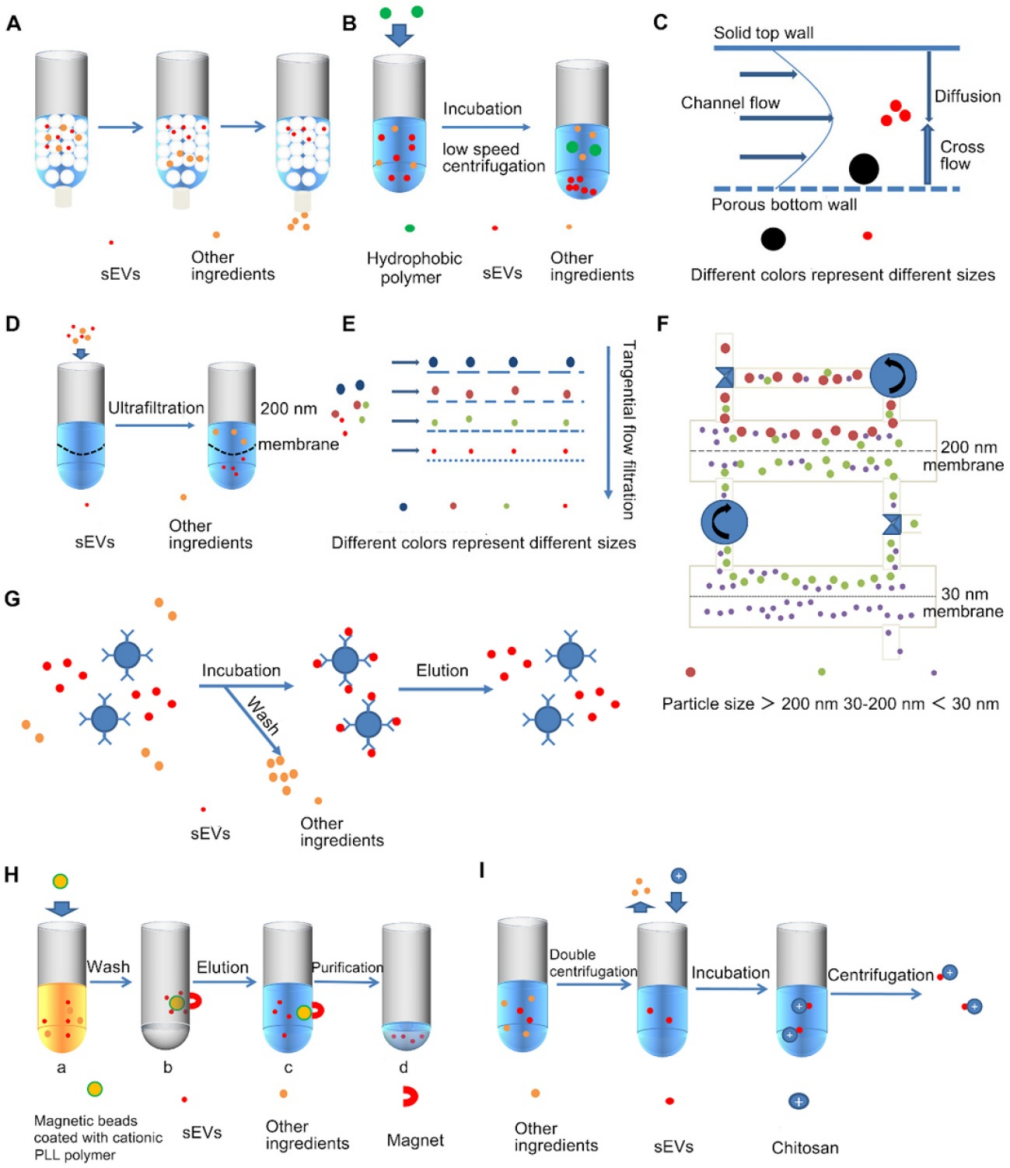
** Simplified diagram of various sEVs separation techniques. A.** Size-exclusion chromatography, **B.** Precipitation, **C.** Asymmetric flow field flow fractionation (AF4), **D.** Ultrafiltration, **E.** Tangential flow filtration (TFF), **F.** Circulating tangential flow filtration, **G.** Immunoaffinity capture technology, **H.** Magnetic bead-based ion exchange technology, **I.** Chitosan based separation techniques.

Size Exclusion Chromatography (SEC) is a widely recognized method that uses polymers to form a porous stationary phase in a chromatographic column. sEVs are separated according to differences in path length of different sized molecules or particles. The path length includes both the excluded volume outside the bead and the included volume that incorporates part of the bead volume (**Figure 4A**). When compared with UC, the physical structure and biological functions of the SEC separated sEVs are more complete 54. SEC can also efficiently separate sEVs from soluble contaminants 40 with simple operation and no additional pretreatments are required, and considering its sample compatibility, SEC is suitable for various biological fluids 55. In addition, sEVs do not interact with the stationary phase during SEC isolation, which reduces sample damage, resulting in relatively high yields 46.

Recent studies report the development of an assay to compare multiple isolation techniques (UC, precipitation, and SEC), and proved that SEC is the best method for sEVs purification from cerebrospinal fluid and plasma 56. However, the narrow application range of size exclusion chromatography makes it unfit for large-volume samples 40. Kaloyan et al found that a higher yield could be obtained by using SEC when compared with UC; however, the purity was relatively low 31. Although SEC can partially remove co-separated contaminants, such as part of HDL and small molecule proteins, it is difficult to remove lipoproteins (Chylomicrons and VLDL) particularly with overlapping sizes 57-59. Some improvements have been proposed, including two SEC columns to separate large exosomes (l-exo), exosomes (m-exo) and small exosomes (s-exo) from human urine samples 59, 60. Guo et al. proposed a simple dichotomic SEC, where they selected the CL-6B column, and performed optimization of the bed volume, raising it from the original 10 mL to 20 mL, and also replaced multiple elution steps with two large elution steps to simplify the complexity of the operation 43. This improvement is more suitable for isolation of sEVs and proteins from FBS, human serum, and FBS-free cell culture supernatants. It has been experimentally demonstrated that this method can improve the reproducibility of applications in clinical settings while obtaining high-quality sEVs with high particle recovery 43. A combination of ultrafiltration and size exclusion chromatography performs well giving both high yield and purity 61.

### Precipitation

Precipitation is a separation method based on the dispersibility of the buffer where the sEVs are located. Hydrophilic polymers, such as polyethylene glycol (PEG), are usually used as a highly hydrophilic polymers that interacts with surroundings to create a hydrophobic microenvironment, thus enabling the precipitation of the sEVs 62, 63 (**Figure 4B**). The specific operations include steps to remove large contaminants, such as cell debris and apoptotic bodies, and precipitation operations. Precipitation has a higher yield than UC, and does not require specialized equipment, and is simple to implement 63. Precipitation also preserves the structural integrity and biological function of sEVs 64, and can fit in a wide range of starting volumes from 100 μL to several milliliters. Notably, precipitation is particularly useful for small-volume samples, and is extensively used for RNA analysis of EV fractions 65. A large number of precipitation-based commercial kits are currently available, such as the Total Exosome Isolation kit (Invitrogen), Exoquick^TM^ (System Biosciences), Exoprep (HansaBioMed), miRCURRY (QIAGEN), ExoGAG (NasaBiotech), Pure Exo (101 Bio), Exosome precipitation solution (Immunostep) and the Total sEVs isolation reagent (Thermo Fisher Scientific) 38. However, it is difficult to separate polymers such as PEG from sEVs, which may affect the results of subsequent research. PEG-separated sEVs are also contaminated with co-precipitated substances, especially some plasma lipoproteins and non-sEVs vesicles, making PEG ineffective for separating plasma 63. Moreover, cell viability was reduced in samples separated using PEG when compared with UC, indicating that the co-precipitated substances may have toxic or antagonistic effects. 22. The contamination of co-precipitated substances can be reduced by adding a high-efficiency pre-filtration step with a 0.22-micron filter or a post-precipitation purification step 66.

### Asymmetric flow field flow fractionation

Asymmetric flow field flow fractionation (AsFIFFF4, AF4) is a technique for separating EV subtypes, as well as sEVs 67, 68 (**Figure 4C**). The AF4 flat channel is composed of two plates, the upper wall of the AF4 channel is a water-impermeable polycarbonate glass plate, and the lower channel plate is breathable, made of porous stainless-steel frit material, with a polyester trapezoidal separator and an ultrafiltration membrane in the middle. Bottom channel plate and ultrafiltration membrane can form agglomeration walls 69. The size-based isolation is achieved by a transverse flow perpendicular to the parabolic flow pattern. The particles to be analyzed flow towards the channel floor or accumulation wall under the action of the transverse flow. At the same time, the particles diffuse to the center of the channel under Brownian motion. Depending on the diffusion coefficient, the molecules are separated into different laminar flows 69, 70. Smaller particles have higher diffusion coefficients and therefore are separated later than larger particles. This technique is a gentle isolation strategy without strong shearing force so that maintains sEVs structural and biological integrity and allows the isolation of EV subtypes. AF4 has now been shown to isolate sEVs from cell culture supernatants and human serum 71. Optimization of the parameters of AF4 such as cross flow gradient, focusing time, sample ultrafiltration conditions, plasma volume, and injection volume, can improve reproducibility and resolution, and separate lipoproteins from EVs 71. AF4 can also be used for resolve the complexity of heterogeneous nanoparticle subpopulations. For example, Zhang et al identified two subpopulations of exosomes and non-membrane nanoparticle “exomeres” by using this method 68. However, this technique requires specific equipment and personnel with expertise to regulate and optimize various parameters (e.g. cross-flow velocity, channel height, and membrane type) 38. Moreover, AF4 is not suitable for large-volume samples, which need to be pre-concentrated by UF, UC or sEVs isolation kits. Combined use of immunoaffinity chromatography (IAC) and AF4 can be employed for the automated isolation of CD9^+^ and CD61^+^ sEVs 72. The IAC-AsFlFFF system provides highly reliable and reproducible separations as the relative standard deviations of EVs yield between fractionation cycles are only 2.9-4.2% and can automatically process up to 18 plasma samples per day 72. Moreover, asymmetric flow field fractionation coupled with UV and multi angle light scattering (AF4 /UV-MALS) can be used to separate sEVs from urine with a high degree of reproducibility, while determining their size, quantity and purity of the isolated urine SEVs, and also enabling isolation analysis of sEVs subtypes 70.

### Ultrafiltration

Ultrafiltration (UF) is an isolation technique based on the size of sEVs 33. UF uses membranes with molecular weight cut-off (MWCO) ranging from 10-100 kDa 59, filtration membranes are usually made of cellulose, polyethersulfone or hydrogenated salts, among which cellulose film is mostly used 38. sEVs from a large amount of raw material are concentrated into a small volume sample so that they can be suitable for subsequent isolation and purification steps 37 (**Figure 4D**). UF is simple to handle with no additional need of special equipment, and can separate sEVs with well-defined particle sizes by adjusting the pore size of the filtration membrane 73. It is reported that ultrafiltration has the highest recovery rates for particles smaller than 100 nm, including sEVs, and it improves sEVs yield and isolation efficiency with a shorter processing time compared to UC 74, perhaps it can be one of the alternative methods to UC 37.

There are also two devices with simple operation and high isolation efficiency named microfilters configured in series 75 and continuous filtration. Tandem configuration filtration uses two filters with different filtration pore sizes in series, a 200 nm filtration membrane on top and a 20 nm one below, leaving large particles (larger than 200 nm apoptotic bodies) in the upper layer, small particles (smaller than 20 nm proteins) in the layer below, while the desired ingredient (sEVs) in the middle layer. Based on the time-consuming continuous filtration operation, “ExoMir™ Exosome Isolation” isolation kit was developed 76. However, non-sEVs still can be co-separated together with sEVs. Particularly, interaction with the membrane as vesicles passing through the membrane can lead to clogging of the pores of the filtration membrane, resulting in a high rate of damage to the filtration membrane and increased cost 22. Shear forces also disrupt the integrity of sEVs 35. Researchers have subsequently developed a tangential flow filtration (TFF) 77, the particles flow along the membrane are parallel to the membrane surface, rather than flow to the membrane or perpendicular to the membrane, which can cleverly avoid clogging problems 38 (**Figure 4E**). The experiments proved that compared with UC-sEVs, the production of TFF-sEVs was increased by 18 times, and its anti-apoptotic effect was also improved 74.

On this basis, Kim et al. proposed a circulating tangential flow filtration (TFF) system (**Figure 4F**) 78, which consists of two membranes with pore sizes of 200 nm and 30 nm connected to a peristaltic pump. This system has higher isolation purity than single cycle TFF. Compared with ExoQuick, it can better ensure the integrity of sEVs structure and biological functions 78. If the pore size of the filtration membrane is adjusted, it is possible to separate particles with a well-defined particle size. Also, Luca Musante et al. proposed a new sEVs isolation technique, hydrostatic filtration dialysis, which can concentrate samples and adapt to large-volume samples. It can also eliminate the influence of soluble protein on purity, and reduce the amount of loss, and the yield is better than UF 79. UF is suitable for application together with other methods, such as UF-LC ultrafiltration combined with size exclusion liquid chromatography, can obtain purer and structurally complete sEVs than UC 41. There is also a sEVs total isolation chip (ExoTIC) based on ultrafiltration technology 80, which is easy-to-operate and high-yield 22. Xiang et al. proposed an ultrafiltration-TiO_2_ series method combining ultrafiltration and phospholipid affinity-based EV isolation. The phospholipid affinity-based method used the specific interaction between the metal and phosphate groups on the lipid bilayer for separation 81. This hybrid method is fast, capable of processing a large number of urine samples and produces high-purity EVs, and can be considered as an alternative method for processing urine samples 81.

### Immunoaffinity capture

Immunoaffinity capture technology is primarily based on sEVs membrane surface protein markers such as CD9, CD63, CD81, CD82, annexins, programmed cell death 6 interacting protein, Rab5, and epithelial cell adhesion molecules 22, 82 (**Figure 4G**). Several immunoaffinity capture-based methods have been developed using microtiter plates, affinity columns or magnetic beads 82, 83. Immunoaffinity capture is especially suitable for isolating EV subtypes based on markers rather than isolating all EVs at one time with a relatively low yield but high purity 38. When studying specific EV subpopulations, after performing UC or SEC to isolate sEVs, immunoaffinity capture can efficiently isolate specific EVs. For example, when conducting immunocapture of melanoma-derived exosomes from plasma, morphologically intact and biologically active sEVs can first be obtained by mini-size exclusion chromatography (miniSEC), and then specific tumor-targeted antibodies, antigen peptide epitope chondroitin sulfate peptidoglycan4 (CSPG4) monoclonal antibody is then used to precisely isolate MTEX 84. Related commercial kits have also been developed, such as the exoRNeasy Serum/Plasma Kit (Qiagen, Hilden Germany), which is widely used to purify sEVs-derived total RNA from serum/plasma. However, due to the low yield and high cost of this technology, it is difficult to apply it on a large scale 37. Notably, while the yield here is low for total EVs, for a particular subpopulation that is isolated, the yield is relatively high 17. Importantly, the specific binding of immunoaffinity capture is hard to reverse, thus affecting subsequent experiments. Use of molecules with reversible binding can avoid such problems. For instance, the use of Tim4 peptide; the specific binding of Tim4 is Ca^2+^-dependent, and it can bind to phosphatidylserine that is specifically expressed on the surface of sEVs, and the Tim4 peptide is immobilized on magnetic beads for isolation. Finally, sEVs can be dissociated from the beads by adding Ca^2+^ chelators 85. A cleavable-linked antibody immobilization method can also be used. Kang et al. propose an sEVs-specific dual-mode immunofiltration (ExoDIF) device that introduces 3,3'-Dithiobis (sulfosuccinimidylpropionate, DTSSP) on the surface of the antibody and immobilizer, this linkage can be cleaved by tris(2-carboxyethyl) phosphine (TCEP) or dithiothreitol (DTT), resulting in the release of the sEVs 39. In addition, Zhu et al. developed a column-based CD9-antibody-immobilized HPLC immunoaffinity chromatography (CD9-HPLC-IAC) technique, which can separate sEVs in real-time from trace serum (40 μL) within 30 minutes 86. The advantages of the method are that it is small scale, high efficiency, and can be monitored real-time. Compared with UC and SEC methods, the contamination of proteins and apolipoproteins from blood is greatly reduced, and the purity is improved 86. At the same time, this method also ensures the integrity of sEVs, and the immunoaffinity method is optimized in all aspects.

### Charge-based separation techniques

sEVs are negatively charged particles varying from their origin cells since different cells have different charges and are highly heterogeneous, which may affect the isolation efficiency 87. Therefore, the distinct methods should be selected by considering different cell sources. A number of methods have been developed based on the negatively charged properties of sEVs.

#### Chromatography-based systems

The chromatography-based systems mainly rely on the interaction between the zeta negative potential of the sEVs membrane and the positively charged anion exchanger, which allows the dissociation of EVs from the positively charged medium by increasing the buffer ionic strength (by introducing a high salt concentration). Applications like anion exchange chromatography (AIEC) involves the use of a monolithic column with quaternary amine functionality (strong anion exchanger) 88 and diethylaminoethyl cellulose resin (weak anion exchanger) 89. This method shows high operability and scalability; however, it is currently mostly used for cell culture medium as many biological fluids contain complex components and charged substances 59.

In addition, a novel chromatographic method, developed by Ken Marcus and colleagues, proposes a separation and purification strategy using hydrophobic interaction chromatography (HIC) which uses a polyester capillary channel polymer fibrous phase 90. Capillary channel polymer (C-CP) fibers show a certain special structure as when packed in the form of columns, the fibers interdigitate to form numerous 1-4 μm channels, providing high permeability for fluid flow 90. It has hydrodynamic advantages combined with a high degree of chemical separation versatility and can also modify the fiber surface to affect high ligand densities for ion exchange (cations and anions) and affinity chromatography 91. Hydrophobic exosome surfaces adhere to the weakly ionized surface of poly (ethylene terephthalate) (PET) fibers, making HIC a selective method for exosome isolation 90. The method has been extended to a more clinically beneficial EV isolation workflow that uses a 1cm C-CP fiber connected to a micro shift tip to allow solid phase extraction (SPE) of EV in a bench centrifuge 92. The sEVs isolated by this method have good integrity, relatively low cost, high yield, and high cost performance compared to differential centrifugation (DC). Most importantly, they have recently discovered that this method can also separate sEVs from LDL, greatly improving the purity 93. In addition, studies have demonstrated that this method can also isolate sEVs from human urine, saliva, cervical mucus, serum, and goat milk matrices, with a high degree of generality. It can be seen that chromatography-based methods do hold great potential for clinical application in the future 94.

#### Magnetic bead-based ion exchange technology

Kim et al. proposed a magnetic bead-based ion exchange technology, ExoCAS-2 (sEVs clustering and scattering), which is a flowable resin that can freely move and adsorb counter-ionic objects in the liquid phase 95. sEVs can be separated by adhering to magnetic beads coated with polycationic polymers. The specific process is shown in **Figure 4H** 95. This method relies primarily on magnetic, particle-based mobile ion exchange resins that can easily isolate high-purity and high-yield sEVs with well-controlled bead size, uniform polymer coating, and magnetic operation, making it highly reproducible and repeatable. It is also scalable for a wide range of sample volumes 95. Moreover, compared with the traditional fixed resin, this flowable resin can reduce the loss of sEVs due to the reduction of the flow rate and the sEVs yield can be improved by optimizing the final washing and elution steps. Experiments show that the highest washing efficiency can be obtained by using buffer solution (pH=6), and the highest elution efficiency can be obtained by using 1M NaCl buffer 95. The method is simple and time-consuming and it is worth of attention that this technique cannot be applied to urine for high concentrations of chloride ions, which affect sEVs capture 95.

#### Chitosan based isolation techniques

A new charge-based method has recently been discovered to separate sEVs from a variety of biological fluids using the polysaccharide chitosan (**Figure 4I**). Chitosan is an alkaline deacetylated derivative of chitin 96, it is a linear cationic polyelectrolyte polysaccharide with biological properties such as biocompatibility, non-immunogenicity, biodegradability and low toxicity 97, 98. Chitosan shows various biological uses in viral infection treatment and bone repair. 99-101. Chitosan can be soluble and protonated in an acidic environment. The high positive charge of chitosan easily attracts sEVs, which carry a net negative zeta potential, ranging from about -10 mV to -20 mV 102, 103. The biological fluid is first subjected to a two-step pretreatment, to remove cells, debris, and large particles. Then adding chitosan into the pretreated biological fluid to incubate the chitosan-sEVs complex. Finally, the chitosan-sEVs complex is centrifuged and subjected to subsequent research 104. Awanit Kumar et al used chitosan to isolate sEVs from cell culture conditioned medium (CCM), human plasma and various body fluids, respectively, and analyzed the proteomics of sEVs in chitosan-isolated CCM, urine and saliva, which in turn demonstrated that chitosan can separate sEVs from biological fluids. Each biological fluid has different physicochemical complexities, indicating the compatibility of chitosan isolation technique in various types of biological fluids 104. Besides, chitosan immobilized on magnetic beads separate sEVs, thus proving the versatility and adaptability of chitosan to sEVs isolation platforms 104.

As a matter of fact, the zeta potential of sEVs changes as the ionic strength of the environment, and the surface charge of chitosan can also change by adjusting its formulation. For example, the acidic formulation of chitosan provides more positive charges than the neutral one. This is also the reason why the chitosan acid formula is more effective in separating sEVs. Given that different biological fluids possess distinct physicochemical properties, such as the protein and urea content, which can affect the efficiency of chitosan for sEVs isolation, it is necessary to select different formulations of chitosan based on the sample origin 104. Chitosan-mediated sEVs isolation uses low-speed centrifugation, which may have little physical damage to sEVs function when compared to ultracentrifugation. As for the comparison with immunoaffinity or antibody-based capturing, chitosan is safer and nearly non-toxic which has been proved by locally use (wound healing) and even diet for decades. In general, the isolation of sEVs from chitosan not only ensures its integrity, but also has a low technical cost, and the most important thing is that chitosan is relatively safe to apply *in vivo* 105, 106, thus making it a fairly promising strategy for sEVs isolation 104.

### Microfluidics

Microfluidic technology integrates sEVs isolation, detection, and analysis in miniaturized chips 107 with the benefits of reduced time, high sensitivity, specificity and high production 108. Besides, not much sample volume and reagents are required to support the whole process and the on-site analytical capabilities make it a user-friendly, clinically reliable and cost-effective choice for sEVs isolation 33. The microfluidic platform is also widely used for DNA, protein and virus isolation 37. Currently, there are two main types of microfluidics based EV isolation strategies, namely, label-based and label-free isolation technologies 109.

#### Label-based microfluidics

**Figure 5:**
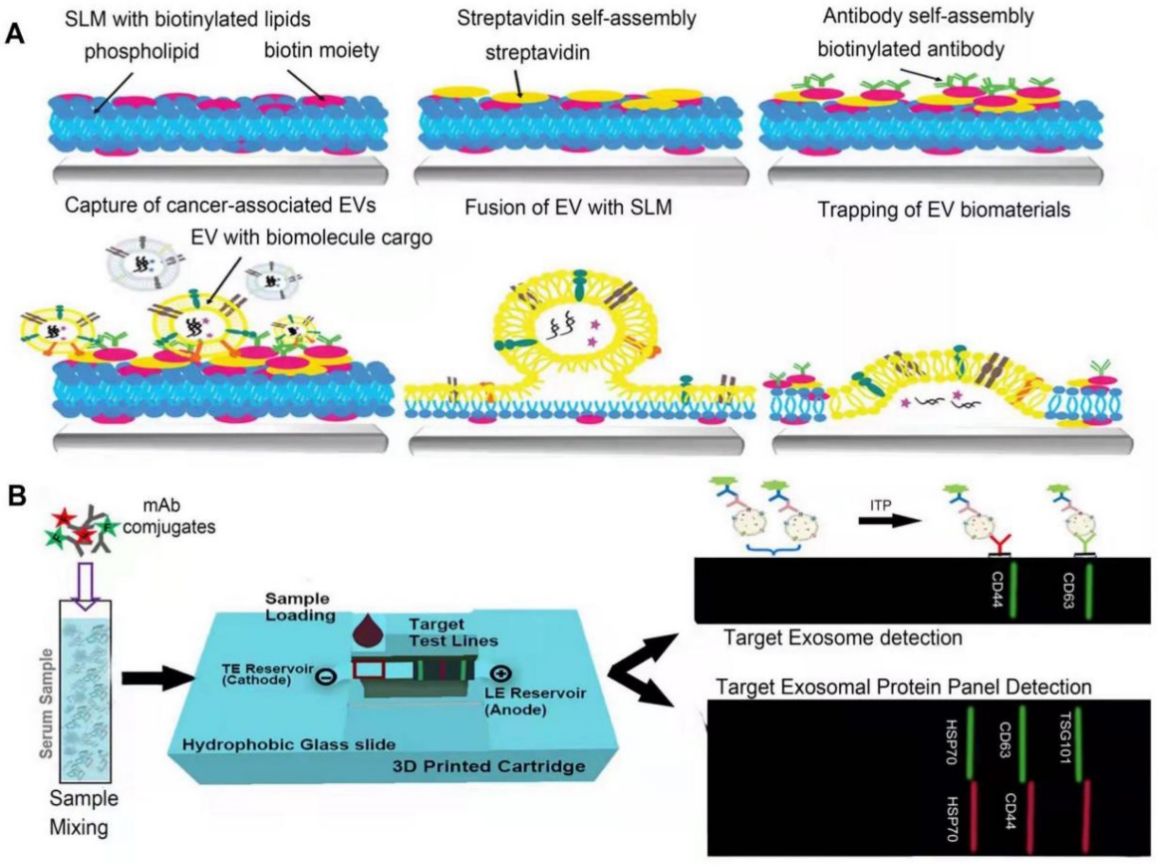
** Simplified illustration of microfluidics methods. A.** Scheme of lipid membranes microarrays. Biotinylated SLM arrays are firstly fabricated using lipid dip-pen nanolithography (L-DPN), overlaid with streptavidin. Then, biotinylated antibodies (ABs) are bound to the SLM array. Finally, the separation can be achieved by exposing the array to a solution of sEVs with the marker of interest. Adapted with permission from 110, copyright 2021 John Wiley and Sons. **B.** A paper-based isotachophoresis (ITP) technology. Place the paper strip on a glass slide with both ends dipped into the container, and then mount the ITP box on a fluorescence microscope stage equipped with a DFC310 digital color camera, below the 4× objective. After loading the sample, a voltage of 150 V was applied to the anode and the cathode can be grounded to start the ITP experiment, monitoring targeted enrichment with labeled fluorescence. Adapted with permission from 114, copyright 2020 Elsevier.

The basic principle of label-based microfluidic technology is kind of similar to that of immunoaffinity capturing. Capturing molecules, such as antibodies and aptamers, can specifically bind to corresponding lipid components or proteins on the surface of EVs on the basis of chemical or physical properties 109. Modified magnetic beads or nanomaterials and antibodies/aptamers immobilized on the surface of microchannels are commonly used for EV isolation 107. Liu et al. proposed a novel EV capture strategy based on dip-pen nanolithography technology, which efficiently isolates sEVs by carrying antibodies that specifically recognize surface markers of sEVs (**Figure 5A**) 110. Furthermore, there are still many label-based microfluidic technologies for isolating sEVs based on specific surface markers 33. The separation sensitivity can be improved by modulating the structure-specific and chemical properties of the microfluidic channel to increase the surface area for the interaction between the microfluidic channel and sEVs. ExoTIC is a novel development that is an exosome total isolation chip device. ExoTIC uses a simple filtration method to isolate intact sEVs in the 30-200 nm size range by a nonporous membrane. The yield of sEVs by ExoTIC yields 4-1000 times higher sEVs than UC 80. Moreover, other researchers also demonstrated that this technology is also a modular platform which can be adapted to a variety of samples and can classify heterogeneous populations of cancer cell line EVs by sizes 80. However, the cost of labeling is relatively high, the labeling operation is complex, and the capturing molecules are also likely to change the physical and biological properties of the sEVs 33, 109.

#### Label-free microfluidics

In order to reduce the influence of labeling on subsequent experiments, label-free isolation strategy has been proposed, which is mainly dependent on the physical properties of sEVs, such as size, electrical properties, and deformability 33. A growing number of label-free microfluidic platforms have been developed, including methods based on sieving, deterministic lateral displacement, field flow, and entrainment fractionation, viscoelastic, acoustic, inertial, electrical, and centrifugal force 38, 109. The greatest benefit of using label-free strategy is to avoid the destruction of sEVs, guaranteeing their structural and biological integrity. Besides, it also simplifies the operational steps to save time and cost, improves application reproducibility and reduces the risk of contamination. On the other hand, label-free isolation cannot completely remove contaminants such as proteins and cell debris. This technology is still in the process of advancing from the micro-scale to the nano-scale, but a combination of technologies provides optimized outcome, such as DLD sorter coupled with a spiral inertial microfluidic sorter in series, inertial microfluidics combined with an integrated membrane filter, or an external dielectrophoretic force, inertial microfluidic sorter combined with an acoustic sorter 109. It is believed that the continuous efforts on microfluidics may lead it to a better future for clinical application.

Acoustic-related microfluidic technology is mainly based on the variant acoustic force received by particles with different sizes 107, 111, 112. For example, Zhao et al. developed a unidirectional disposable acoustofluidic platform based on a unidirectional interdigital transducer (IDT) 113 with high isolation efficiency and versatility. Electrically related microfluidics is developed on the basis of electric field strength and particle charge, such as the employment of electrophoresis (**Figure 5B**) 114 and dielectrophoresis (DEP) 115. The centrifugal microfluidic system is a lab-on-a-disc integrated with two nanofilters (Exodisc), which mainly perform separation based on particle size, and can fully automate the separation of EVs in the size range of 20-600 nm in a short time 116. Inertial isolation 117 takes the advantage of inertial migration, where the particle size determines inertial resistance and viscous resistance, so that isolation can be performed according to particle size. Viscoelastic isolation can be achieved by the size-dependent elastic lift in viscoelastic fluid media 109, 118.

### Synthetic peptide (Vn96) based isolation method

Anirban Ghosh et al. designed a series of peptides (venceremins, or Vn peptides) and discovered a new class of peptides (Vn96), which showed nucleotide-independent specific affinity for typical heat shock proteins 119. Various experiments demonstrated that Vn peptides can specifically and efficiently capture HSP-containing sEVs from cell culture growth media, plasma, and urine. sEVs were first isolated from the samples by ultracentrifugation, the isolated samples were incubated with Vn96 overnight at 4 °C with rotation, then centrifuged at 17,000 × *g* for 15 min at 4 °C, and finally washed three times with PBS 119, The characteristics of the final product were similar to those obtained from UC isolation, which proved the reliability of the technique. Irene V Bijnsdorp et al. found that this method is more convenient, time-saving and has higher yields than UC 120. Notably, Vn96 peptide can be combined with HSPs from various species; therefore, this method is not only suitable for human body fluid samples (such as plasma and urine) 121, but also suitable for animal samples (mouse and dog plasma), making it applicable in animal experiments for basic medical research. Overall, the Vn96 peptide isolation method can isolate sEVs of clinical value and outperform current isolation methods in terms of efficiency, cost, and platform versatility, and can also be applied to basic research in animal models 119.

### Other new isolation strategies

#### EXODUS

**Figure 6:**
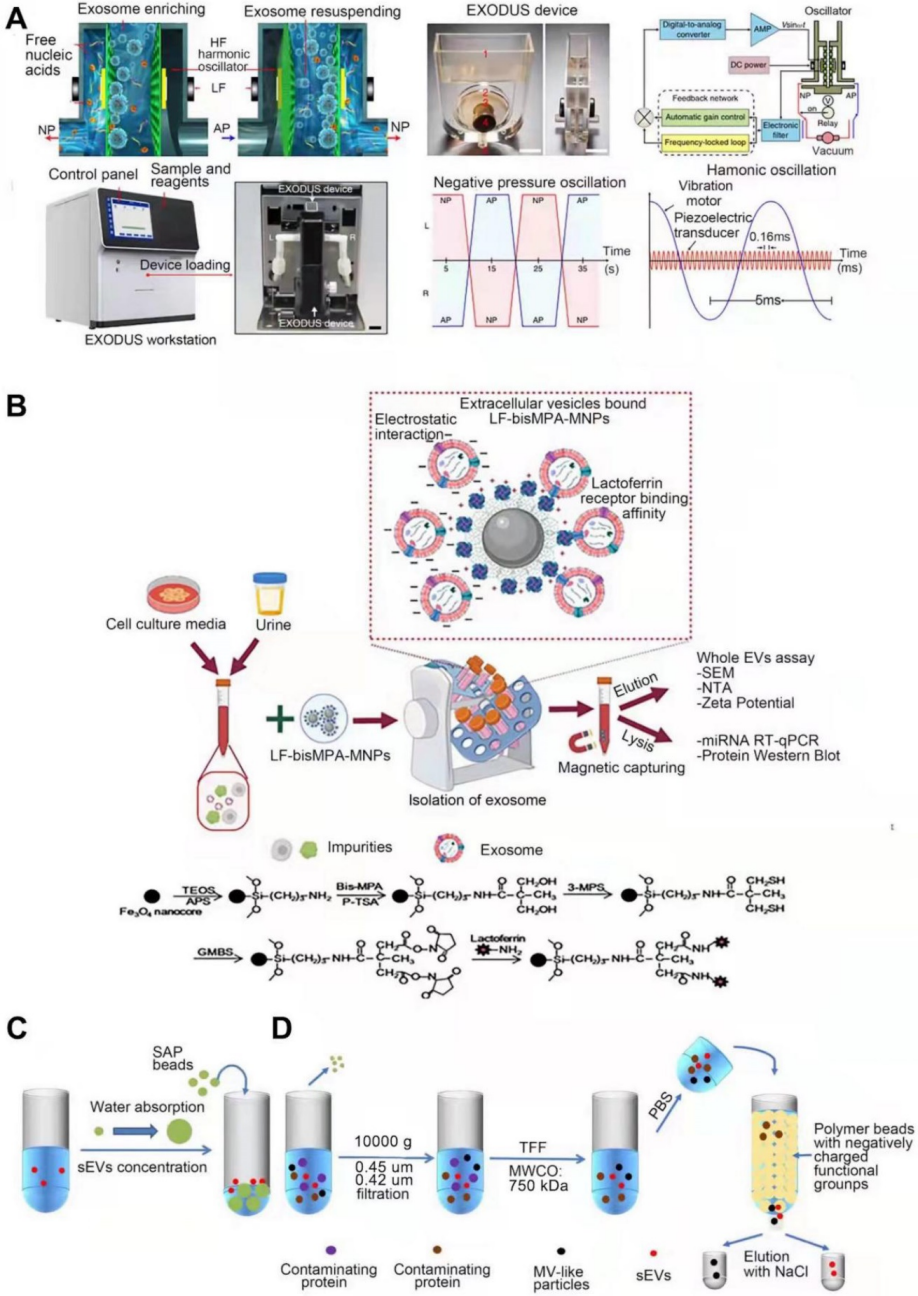
** Simplified illustration of sEVs emerging isolation techniques for sEVs. A.** EXODUS. Adapted with permission from 122, copyright 2021 Springer Nature. **B.** Chimeric nanocomposites. Adapted with permission from 123, copyright 2022, John Wiley and Sons. **C.** SAP-based technology. **D.** Anion exchange-based method.

Liu et al. developed a technique “EXODUS” (**Figure 6A**) that could automatically isolate sEVs from various biological fluids without labeling 122. With negative pressure oscillation and a dual coupled oscillator that vibrates the membrane, highly efficient isolation of exosomes can be achieved. The two coupled oscillators generate a double frequency transverse wave across the membrane, making EXODUS superior to other isolation techniques in speed, purity, and yield. Periodic negative pressure oscillations (NPOs) in the device can effectively avoid the aggregation of particles such as contaminant proteins and vesicles, on the membrane, along with the possible clogging of the pores in the nanoporous membrane by these particles 122. In addition, during the isolation process, due to the negative pressure, the integrity of the physicochemical properties of exosomes are able to be reserved. Different harmonic oscillators, such as NPO alone or combined lactoferrin, can be set up on the EXODUS unit to combine different harmonic oscillators. Liu and colleagues also applied EXODUS to plasma, urine, culture medium, and even tears, demonstrating the adaptability of this technology to different types and volumes of biological fluids 122. Overall, this technique compares favorably with others in terms of speed, purity, and yield, and has no limitation on specimen volume, enabling isolation and purification of SEVs in a noninvasive and cost-effective manner 122. However, the current EXODUS platform is limited to single-channel isolation. Large-scale biological research will be facilitated by implementing a series of EXODUS devices with automated reagent distribution and sample collection for multi-sample processing 122. The smaller nanopore size of EXODUS can be further studied to specifically isolate more sEVs subtypes. Integrating EXODUS with downstream assays can also be explored. For example, Liu Fei et al combined EXODUS with MALDI-TOF MS to obtain highly sensitive proteomic fingerprints of intact sEVs from 20 μL of human plasma. They also applied EXODUS to isolate sEVs from urine samples of kidney and bladder cancer patients for transcriptional profiling, which demonstrated the feasibility of the clinical application of EXODUS 122.

#### Chimeric nanocomposites-based technology

Chimeric nanocomposites of lactoferrin conjugated 2,2-bis(methylol) propionic acid dendrimer-modified magnetic nanoparticles (LF-bis-MPA-MNPs) are reported to efficiently separate sEVs from human urine 123 (**Figure 6B**). This method is mainly based on physical absorption, electrostatic interaction and biorecognition of sEVs 123. The N-terminus of LF is cationic and hydrophobic, maintaining a net positive charge at high pH level (8.0-8.5) 124 while the phosphatidylserine (PS, a negatively charged lipid) of sEVs provides a net negative charge 125, allowing sEVs to bind to lactoferrin (LF). LF also interacts with glycosaminoglycans and chondroitin sulfate proteoglycans 126, the van der Waals force providing the driving force for physical absorption. GAPDH expressed on the surface of sEVs acts as a receptor for LF 127. These three strategies enable efficient isolation of sEVs, after which the LF-sEVs complex can be dissociated by eluent buffer (pH~10.6) to further purify sEVs 128. sEVs isolated from conditioned medium or urine are subsequently subjected to following identification. Compared to the existing methods, this method has better isolation speed, efficiency, yield and purity, and can also maintain the integrity and biological functions of sEVs, with no requirements for expensive equipment is required 123. However, when this method is selected to isolate sEVs, it is difficult to completely distinguish them from other types of extracellular vesicles.

#### SAP -based technology

Yang et al. developed the first single step, equipment free method of sEVs concentration by using high water absorption polymer (SAP) beads (**Figure 6C**) 129, which are able to absorb small molecules including water through nano-sized channels while sEVs are excluded 130, 131, leading to the final concentration 129. In addition, SAP can absorb several contaminants, such as proteins, thereby improving the purity of sEVs. During this process, no external forces act on sEVs thereby maintaining their integrity. This method is mainly used for concentration, and the isolation purity is not that high. It needs to be used in combination with other high-efficiency isolation techniques, such as SEC. Experiments have shown that this method is versatile for various biological fluids and media, such as urine and plasma 129. It can also adapt to samples of different volumes and concentrations by adjusting the absorption time and the concentration of SAP, and the integrity of the sEVs remains intact during this process. This method can also be applied for the detection of biomarkers transported by sEVs. For example, Yang et al. found that in the process of using nanoscale oligonucleotide probes to detect sEVs miRNA 132, SAP can absorb the free probes, amplifying the detection of probe-miRNA hybridization signal, reducing background signal and improving detection sensitivity 129. Further, this method can be widely used in liquid biopsy to improve its sensitivity of biopsy and facilitate disease diagnosis 129. The advantages, such as being simple-to-operate, cost-effective, and equipment-free, make SAP-based sEVs isolation hold great promise for popularization and application 129.

#### Anion exchange-based method

A new separation method based on anion exchange column chromate graphy was reported (**Figure 6D**) 133. This method is used mainly to isolate and purify sEVs and other EVs by using anion exchange column chromatography followed by elution at high (0.3 M ~ 0.5 M) and low (0.15 M ~ 0.3 M) NaCl concentrations, respectively. The protein removal rate of this method was over 99.97% 133. The principle of anion exchange resin separation is described in the “Charge-based separation” section above. This method can efficiently isolate sEVs, and the selection of elution conditions as the key to step. sEVs present weaker affinity for phosphatidylserine (PS)-binding proteins AnnexinV and lactadherin than other EVs 134, and have low surface sialic acid content 133. These factors allow sEVs to have a relatively weak negative charge, thereby distinguishing them from other EVs particles. The EVs obtained by elution at two different NaCl concentrations were analyzed by proteomics, DNA, morphological size, miRNA distribution, zeta potential values, target cells, and surface glycosylation 133. The results indicated that the particles obtained at low NaCl concentrations (0.15 M~0.3 M) were sEVs for they have sEV-specific proteins, including late endosome-related proteins, integrin and rab family of proteins, as well as functional miRNAs 133. They also show biological activity to prevent tumor metastasis by depleting the mesenchymal cell population in primary tumor lesions 135. The particles obtained at high NaCl concentrations (0.3 M~0.5 M) were microvesicle (MV)-like particles 133. This isolation method can produce sEVs with high purity on a large scale without affecting their biological activity, providing an efficient method for the research and clinical application of sEVs.

### Cocktail strategy

**Figure 7:**
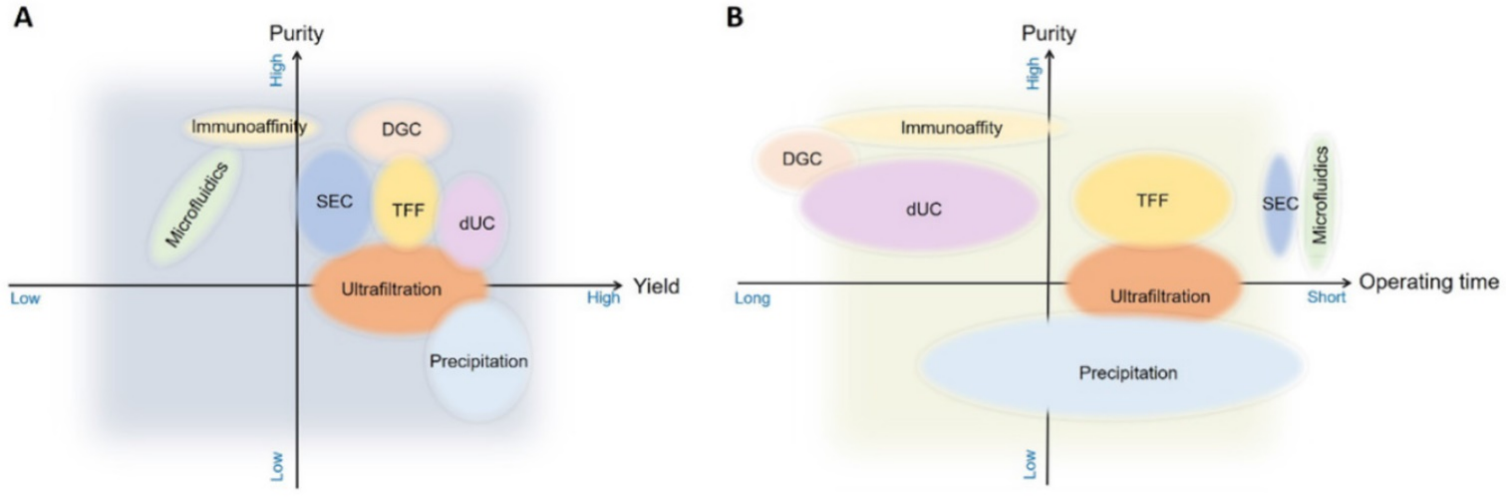
** The methods used most often for sEV isolation are illustrated comparing their performance in purity, yield, and processing time.** DGC, density gradient centrifugation; SEC, size exclusion chromatography; TFF, tangential flow (cross-flow) filtration; dUC, differential ultracentrifugation.

**Figure 8:**
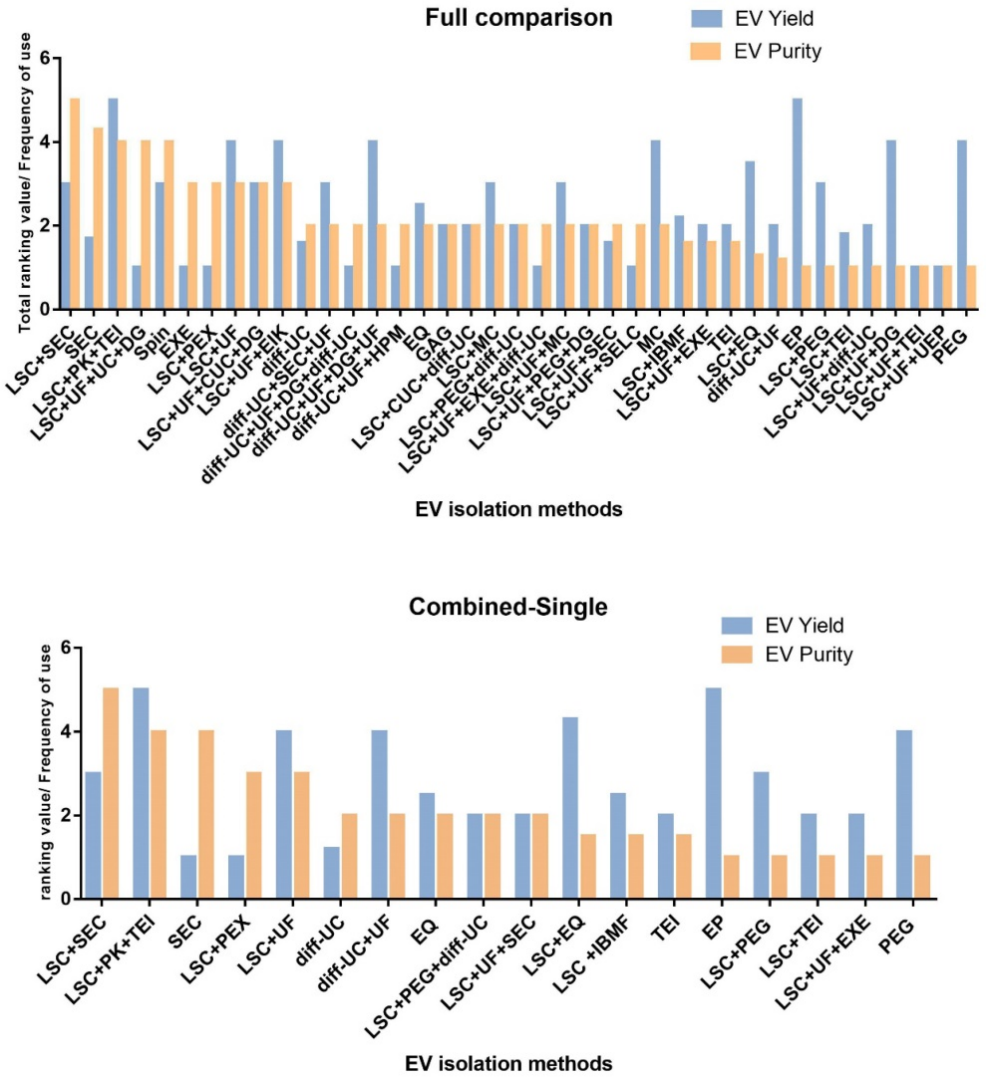
** Comparison of sEVs isolation methods in terms of purity and yield, sorted from highest to lowest purity.** Adapted with permission from 38, copyright 2021 Elsevier. A. Comparison of all sEVs isolation methods, B. Comparison of combined sEVs isolation methods and single sEVs isolation methods. (diff)UC: differential ultracentrifugation, DGUC: density gradient (ultra) centrifugation, CCS: cell culture supernatant, EIK: Exosome isolation kit Pan (immunoaffinity based on CD9, CD63 & CD81), CUC: cushioned-density (ultra)centrifugation, EP: Exoprep (precipitation method), EXE: exoEASY, EQ: Exoquick (precipitation method), GAG: ExoGag (glycosaminoglycan precipitation method), HPM: Heparin/polymer coated microspheres, IBMF: Immunity-based microfluidics (antigen: annexin), LSC: low- speed centrifugation (< 80,000 x *g* regardless of time), MC: miRCURY (precipitation method), SEC: size-exclusion chromatography, PEG: polyethylene glycol (precipitation method), PEX: Pure Exo isolation kit, PK: proteinase K treatment, SELC: size-exclusion liquid chromatography, TEI: Total exosome isolation kit, UEP: Urine exosome precipitation (and RNA isolation) kit, Spin: EXOspin (SEC plus precipitation method), UF: ultrafiltration.

Single methods for sEVs isolation show great variability in many aspects, particularly in yield and purity (**Figure 7**). A cocktail strategy employs optimal combination of methods by sharing their complementary advantages in order to achieve the purpose of high yield and high purity. For example, when sEVs are obtained with centrifugation, which is based on physical properties such as density, co-isolation of substances overlapping with sEVs in terms of density and other physical properties cannot be avoided. Therefore, a complementary method can be used to sequentially remove the co-separated contaminants. It has been shown that the combined isolation strategy is clearly superior to individual isolation methods in terms of achieving higher purity and yield. Size exclusion chromatography is recommended as an initial step followed by low speed centrifugation, the combination of these two methods can achieve the highest sEVs purity while maintaining a reasonable sEVs yields (**Figure 8**) 38. Combinations can also be selected according to different research objectives. Despite the fact that the combinatorial method is suitable for sEVs isolation from highly heterogeneous and complex biological materials and improves the yield and purity, the resulting operation time is relatively long and large samples volumes may be required. These limitations imply that the combinatorial strategy is more likely to be used for cell culture supernatants, but might not be suitable for clinical research and small sample volumes, such as the samples for non-invasive liquid biopsy 38.

## Challenges and outlooks of sEVs and their isolation techniques

### Unsolved problems with respect to sEVs

Despite the remarkable application prospects, there are still questions that are worth discussing. Intrinsic problems of sEVs, such as their heterogeneity in physico-chemical properties, are not well understood and can derivatively influence the choice of different isolation techniques. It is also hard to say which method is the best option since there are too many measuring indicators to be measured, such as yield, purity, and isolation efficiency. From what we've seen so far, choice of the most appropriate methods must be based on each specific research requirement, application scenarios, and sample types. A cocktail strategy by using multiple isolation methods together to improve the purity and/or production is also recommended (**Table 2**).

#### What are the possible causes of the functional heterogeneity of sEVs?

The heterogeneity of sEVs is reflected in their physical properties and biological functions. The biological function of sEVs can be cognized as the effect exerted by the cargo-mediated information exchange between cells or organs 145, which implies that many factors, such as the originating cell, the target cell, as well as the microenvironment, jointly shape the sEV functions 19. For example, it is possible that the same group of sEVs have different roles in different target cells. MSC-derived sEVs promote cell growth in diabetic foot 146 while inhibit cell proliferation in lung cancer 147. The function of sEVs is also affected by the body's microenvironment 148. Patient-derived sEVs are usually shown to carry disease markers, pro-inflammatory factors and generate other destructive effectors, while sEVs derived from healthy people can reduce inflammation, be antioxidant and present other protective effects 148. sEVs functions may also depend on their origin cells 19, possibly due to their distinctive biogenesis and/or content loading mechanism in mother cells 149, resulting in differences in cargo composition of sEVs. Cancer and non-cancer cell-derived sEVs showed a different expression level of HSP70 on surface membrane 150. However, there are currently no specific markers to distinguish sEVs from different cell types from the same individual; sEVs from mixed origin existing in plasma are hard to be precisely separated 151. The functional heterogeneity of sEVs may also be affected by factors that are even currently unknown. More efforts should be made to discover the mechanism that is responsible for the functional heterogeneity, based on which, more accurate isolation techniques could be developed.

#### How to identify EV subtypes?

As mentioned above, currently EV subtypes are mainly classified according to their physical properties (size), biological origin, or their surface markers. The currently reported subtypes are as follows, exosomes (40-160 nm, marker proteins are ESCRT complex proteins and CD63), microvesicles and oncosomes (50-10,000 nm, marker proteins are Annexin A1 and ARF6), migrasomes (500-3000 nm, marker protein is TSPAN4), secretory autophagosomes/amphisomes (size not determined, marker protein is LC3), exomeres (<50 nm, marker protein is unknown), retroviral-like particles (size not determined, marker proteins are Arc1 and Arc2), exophers (1,000-10,000 nm, with labeled protein as Phosphatidylserine, LC3, and Tom20), apoptotic bodies (50-5000 nm, with labeled Phosphatidylserine) 152. Among them, exosomes, microvesicles (MVs) and apoptotic bodies are the three subtypes that have been thoroughly studied. MISEV2018 states that the subtypes of EVs can be classified according to their size (e.g., by filtration, which must be combined with another method, such as SEC, to eliminate non-EV components), density, their surface protein, sugar, or lipid composition (immunal or other affinity separations, including flow cytometry of large particles) or other biophysical properties such as surface charge 17. However, the analysis of EV subtypes remains a great challenge due to the high heterogeneity in their physical properties, composition, and biogenesis. For example, research showed that the presence and abundance of tetraspanins (CD9, CD63, and CD81) on the surface of exosomes from different cells are heterogeneous 153. This also shows that transmembrane proteins such as CD9 may not be effective markers for sEVs recognition when performing isotype analysis of EVs secreted by different cells 153. Studies have shown that in addition to the above subtypes, there are still a large number of particles whose characteristics are unclear. For example, proteomic analysis has reported a high degree of molecular diversity. Purified EVs from a single cell source contains more than 1000 protein signals, which indicates that there are still many undetected EV subtypes 154. Since the resolution thresholds are lower than the standards followed by most optical imaging methods, these undetected subtypes are not easily characterized and identified 155. Choi et al. found that nano-flow cytometry combined with high-resolution microscopy can improve the resolution of EVs subtypes 155. Other researchers proposed an optimized isolation characterization method, an ultracentrifugation-hollow-fiber flow field-flow fractionation orthogonal approach, which has promising results for purification and differentiation of EVs subtypes. The specific operations are as follows: firstly, large EVs and small EVs are distinguished by differential ultracentrifugation, and then these subgroups are analyzed by the HF5 method using UV, fluorescence and multi angle laser scattering as detectors. sEVs are further separated by density gradient centrifugation (DGC), and then analyzed by HF5 multiple detection. The density-dominant isolation principle of DGC is orthogonal to the hydrodynamic radius-dominant isolation principle of HF5, and two-dimensional isolation can be obtained when these two techniques are used together; the size, density, and composition (protein and nucleic acid) of EVs subtypes can be analyzed more comprehensively, and new methods for better purification and localization of EV subtypes can be developed 156. Identifying EVs subtypes is of great significance for developing isolation techniques and improving the accuracy of downstream experiments.

### Challenges and outlooks of sEVs isolation techniques

#### How to ensure the structural and biological integrity of isolated sEVs?

Certain existing isolation techniques inevitably destroy the structural and biological integrity of sEVs, which may largely be due to external force or properties changing caused by microenvironment, such as co-separated substances. Regarding external force, in the process of ultracentrifugation, sEVs are affected by the external force of high-speed rotation 41, and in the process of filtration isolation, extrusion through the filtration membrane can cause the mechanical damage to the extract 157. The way to reasonably avoid external force injury is to adjust and select the appropriate external force. For example, when tangential flow filtration is used for ultrafiltration separation, if the transmembrane pressure is well controlled, the external force can be reduced. Alternatively, one could use method that are not conducive to external forces, such as SEC which depends on natural gravity-based isolation 107. The biological functional integrity of sEVs may be affected by the physicochemical properties of the microenvironment in different biological fluids or by substances that specifically bind to sEVs. For example, in immunoaffinity centrifugation, antibodies that specifically bind to sEVs, the non-neutral pH and non-physiological elution buffers used to elute sEVs during manipulations may compromise the integrity of the biological properties 46. A possible solution to this problem is to use substances that are easy to separate, such as Ca^2+^-dependent Tim4 protein 85. In addition, the polymers used in the precipitation process also have an impact on the biological integrity of isolated sEVs. The selection and improvement of isolation methods are critical for subsequent research and observations.

#### How to ensure the purity of the isolated sEVs?

The low isolation purity of sEVs is mainly caused by the large number of co-isolated contaminants. In particular, the co-isolated non exosomal functional vesicles will affect the accuracy of subsequent experiments 46. If the isolation purity of sEVs in reports is insufficient, then the research results should be carefully verified. Meanwhile, the safety of clinical applications remains to be confirmed. There are also a large number of co-isolated lipoproteins 158 that share overlapping characteristics with sEVs in terms of density, size and lipid content, and their numbers are far more than sEVs; therefore, it is difficult to separate sEVs from plasma without lipoprotein contamination. Challenges still remain in the previously proposed concepts of liquid biopsy 18. In addition, highly heterogeneous biological samples contain substances, such as albumin, casein, and Tamm Horsfall protein 38, that overlap with sEVs in terms of physical biology, which may affect the purity of the isolated sEVs 159, 160, To improve the isolation purity, the primary problem to be solved is to make EVs classification clear, clarifying the specific differences between sEVs and other extracellular vesicles and non-vesicle components in terms of physicochemistry, such as size, density , surface protein, sugar and lipid components, and surface charge 17, which can provide a specific theoretical basis for the development of high-purity isolation technology. For example, the current immunoaffinity technology selects appropriate antibodies or ligands to specifically bind to sEVs based on their surface-specific markers to ensure that their purity can be guaranteed 38. However, according to MISEV2018, it is currently impossible to achieve high purity and high yield at the same time. Perhaps these two parameters can be achieved by using combined methods, such as SEC combined with low-speed centrifugation, to obtain sEVs with suitable purity and yield.

#### How to improve the reproducibility of sEVs isolation methods?

Favorable reproducibility is when every single operation is able to maintain consistency in the quantity and quality of isolated sEVs. The most commonly used ultracentrifugation method currently lacks reproducibility, and the isolated product is affected by the inclination angle of the centrifuge, centrifugation time, sample preparation, and various culture conditions 161. AF4 is an isolation technique with relatively high reproducibility, but when the operator optimizes the parameters of AF4, such as cross-flow gradient, focusing time, sample ultrafiltration conditions, plasma volume, and injection volume 71, human errors and variabilities might still challenge repeatability. We hold the opinion that factors such as the complexity of the operation and the use of additional isolation reagents may lead to variable results. Therefore, automated and easy-to-operate isolation techniques are most likely to guarantee repeatability; the EXODUS technology introduced above may have a high degree of repeatability 122.

#### How to reduce storage-induced damage to sEVs, thereby improving the possibility of clinical application?

The current sEVs storage techniques may cause damage to the concentration, content, and integrity of sEVs, affecting the accuracy of subsequent research and the possibility of clinical application. Most people currently consider 4 °C for short-term 162 and -80 °C for long-term as an acceptable way to store sEVs 138, 163. The effect of different temperatures on the quality and stability of sEVs is unavoidable 164. At 4 °C, sEVs may attached to the tube wall, resulting in a decrease in the number of particles, where smaller sEVs are lost, resulting in an increase in concentration 138. In addition, the contents of sEVs change as total RNA may be lost, and most proteins are also degraded, with the surface-associated proteins (HSP70, CD63, and CD9) altered 165. Storage at -80 °C is the traditional method recommended in the MISEV2014 guidelines, but MISEV2018 does not give a standard storage strategy as it does affect sEVs in this case while the impact is unclear 2. It has been experimentally demonstrated that storage at -80 °C leads to a time-dependent decrease in sEVs dose and purity, an increase in sEVs size and size deformability, and a decrease in zeta potential 165, It also resulted in altered sEVs content, decreased RNA, and decreased levels of surface marker proteins (Alix, HSP70, and TSG101) 166. Besides, cryopreservation produces ice crystals, which disrupt lipid membranes, leading to the release of sEVs contents, resulting in the loss of biological function of sEVs 164. Additionally, freeze-thaw cycles reportedly lead to a decrease in sEVs after the first cycle and an increase in particle size with cycling, and fusion of sEVs was found using flow cytometry 2. However, sEVs stored in semen were not affected 167, which indicated that sEVs from different sources had distinct adaptability to different storage methods, thus increasing the difficulty of standardizing sEVs storage methods. In order to alleviate the effect of freezing on sEVs, researchers have proposed mitigation methods, such as the most commonly used methods of slow freezing and vitrification. Among them, vitrification is a rapid freezing method in which a bulk solution of cryoprotective agents (CPAs) is cooled below the glass transition temperature to avoid producing ice crystals, thereby forming an amorphous matrix, which can also be defined as a very cold viscous liquid. These approaches use low-toxicity CPAs, such as trehalose and dimethyl sulfoxide (DMSO) 168, to protect sEVs 164 and reduce ice crystal formation by increasing the total concentration of all solutes in solution 169. Lyophilization is another technique that removes water from frozen samples by vacuum sublimation and desorption 170. When combined with CPAs, lyophilization can improve the stability of sEVs. However, recently, Stefano Geliber and others have compared different storage strategies and showed that even the aforementioned improved method still lead to the change of the yield, size and charge of sEVs after 4 weeks and 8 months, the integrity will be destroyed, and there is no improvement compared with simple storage at -80 °C 2. However, lyophilization and addition of CPAs over a short period of time (hours) presents a significant improvement. Certainly, the two methods are incomparable since the storage time is different. To sum up, there is no perfect storage method to protect sEVs currently, so fresh samples should be used as much as possible. According to the prevailing consensus, if storage is necessary, it is recommended to store sEVs at -80 °C for a short time in a biological matrix instead of storing separate sEVs 2.

### Possible problems in future applications

#### What are the effects of dosage and mode of administration on biosecurity? How to ensure the safety in clinical applications?

Given that it is difficult to re-separate EV subtypes with the size of 50-150 nm using current technologies, and that the functions of specific subtypes are not well understood, it is difficult to ensure the safety of applying sEVs in the clinic. It is reported that when sEVs are applied systemically, some of them may induce apoptosis of certain cells in the body, while some may induce survival, and some others may induce immune regulation 19. Therefore, it is possible to generate side effects beyond the target organs. Local application may help in avoiding effects on other cells. However, new problems are raised as although local application is obviously safer than systemic application, sEVs are easily to be removed with either treatments within a short time 171, 172. Furthermore, irrespective of the systemic or local treatments, the injection dosage of sEVs should be carefully considered as different doses may cause different biological effects. For example, studies have shown that when sEVs are used to treat neurodegenerative diseases, lower doses of sEVs exhibit neuroprotective effects, while high doses may be harmful to the neurons 173. Factors such as the cell source of sEVs, the mode of administration, and the purpose of application should also be considered to determine the dose. Dhanu et al. carefully summarized the current sEVs dosage according to different isolation methods (purified or not), measurement methods (protein amount/number of particles), experimental animals (rat, mouse and pig), route of administration (systemic/local), application purpose (cardiovascular, neurological, inflammation, and cancer) and clinical trials 3. In short, the body's scavenging effect on sEVs and their heterogeneity enhances the difficulty in determining the precise application dose 148, which requires more efforts in future studies.

#### How to solve the problem of rapid clearance of sEVs by the body and improve their bioavailability?

Most systemically injected sEVs are rapidly taken up by macrophages in the reticuloendothelial system and rapidly cleared from the body. This phenomenon is ubiquitous and unaffected by the mode of delivery or the cell of origin 172. However, topical application provides hope 172. In order to improve the half-life of sEVs, the concept of engineered sEVs has been proposed: a sustained delivery system of sEVs designed by using biomaterials, which could prospectively improve the bioavailability and the ability of sustained delivery of sEVs. The best-studied and most commonly used biomaterial is hydrogel, which is a cross-linked three-dimensional hydrophilic polymer network structure. The polymers used to make hydrogels are divided into natural (hyaluronic acid, chitosan, and gelatin) and synthetic (PEG, PLGA, pHEMA) polymers. Natural materials are usually biodegradable and highly biocompatible, with similarities to ECM, while the synthetic ones are highly tunable in terms of structure, mechanical strength, and degradation rate 174. sEVs can be encapsulated into hydrogels in different ways, one of which is to incorporate sEVs into hydrogels by mixing sEVs with polymers in solution form and simultaneously with a cross-linking agent 175. This technique enables *in situ* gelation, allowing direct injection of hydrogel components (solvent-based polymers + exosomes + cross-linkers) at the desired site 175. Chenggui Wang et al. developed an injectable, self-healing and antibacterial peptide-based FHE hydrogel (F127/OHA-EPL) and applied this technology showing good effects on repair 176. The injectable delivery of the hydrogel is beneficial for its minimally invasive drug delivery to the treatment site, and it has been experimentally demonstrated that the release of sEVs can be regulated by changing the physical structure of the hydrogel to suit different needs 177. In summary, the hydrogel's degradability, tunability of physical structure, and *in situ* gelation make it an sEVs delivery system that can be tailored to specific application needs 175. Hydrogel continuous delivery system can also prevent the rapid clearance of sEVs, improve their bioavailability, and can achieve maximum biological effects with a small number of sEVs. However, it also has the disadvantage of not being appropriate for all applications. For example, the cost of natural polymers is relatively high, and some synthetic polymers that are insoluble in water (such as pHEMA) usually requires the use of strong organic solvents during processing, which may damage the structural integrity of exosomes and reduce sEVs content during mixing 175. In addition, some hydrogels may solidify at a certain temperature and pH, blocking the injecting needle, and the kinetic release condition cannot be determined *in vivo* 175.

## Potential for clinical application of sEVs

### sEVs application in diagnostics

At present, the diagnosis methods for many diseases are still invasive. For example, when using pathological biopsy to judge benign and malignant tumors, may often causes damage to the body and is not able to monitor disease status in current time. sEVs has been developed as a new strategy for the non-invasive liquid biopsy 178. The qualitative (and) or quantitative change of sEVs content is the basis for its use as a marker in disease diagnosis. The numbers of sEVs usually abnormally accumulated in circulation and contents differs from that of healthy status. These changes can be detected by different modern techniques. For example, when the cargos such as proteins, lipids and metabolites are used as disease markers, mass spectrometry can be used to qualitatively analyze the isolated products at the early stage of isolation as well as to determine their types and sources 94, 179. Some more advanced techniques have been developed, for example, Matrix-assisted laser desorption/ionization mass spectrometry (MALDI-MS) are developed to assess vesicle enrichment 17. High-Performance chemical isotope labeling nanoflow liquid chromatography-mass spectrometry (CIL nLC-MS) was developed to analyze the metabolomics of sEVs for more sensitive and rapid detection 180. More importantly, cargos conveyed by sEVs can be maintained and enriched in relatively good quantity, stability as well as integrity 19 and differs depending on donor cells 181. Many studies have shown that cargos such as proteins and miRNAs in sEVs are mostly up to the parental cell types 182, for example, adiponectin can be a specific marker for adipose tissue-derived sEVs 183. Surface proteins of sEVs can also be tissue-specific, for instance, cancer cell-derived sEVs show tumor-associated specific proteins on the surface membrane 184. In this case, sEVs can be quantitatively altered, cargo-protected, tissue-specific and reflect the pathological and physiological state of the body, making them ideal candidates in diagnosing and monitoring diseases.

Many studies prove sEVs as diagnostic markers in early phase of diseases and can be used to reflect disease staging or the severity degree, for example, Jia et al. demonstrated that sEVs derived GAP43, neurogranulin, SNAP25, and synaptic marker protein 1 can be used for predicting the occurrence of Alzheimer's disease 5 to 7 years before cognitive impairment 185. Other researchers found that compared with healthy children, the serum sEVs miR-21-5p increased threefold in children with new-onset type 1 diabetes, which may be helpful for the diagnosis of type 1 diabetes 186. There are also related clinical phase 1 trials to evaluate the role of sEVs in the diagnosis of type 1 diabetes (NCT 04164966). Ling et al. found that the serum levels of miR-126 and miR-21 in patients with acute coronary syndrome (ACS) were significantly higher than those in healthy controls, and by Gensini score, they were also positively correlated with the degree of coronary stenosis in patients 187. And related clinical phase 1 trials are underway (NCT04127591). In addition, many new methods have been developed to specifically capture and detect sEVs for disease diagnosis. For example, Chen et al. developed a DNA cascade reactor, which can quickly realize the signal amplification detection and precise *in situ* imaging of miRNA in exosomes, and can also realize the tracking of exosomes in living cells 181. Liu et al. developed an “open” fluorescent aptamer sensor platform to evaluate sEVs surface proteins, and its sensitivity can be 97.37% in distinguishing benign and malignant breast cancer 184. Even some sEVs based commercial kits has been developed to facilitate cancer diagnosis, which indicates great potential as non-invasive liquid biopsy markers for clinical and business application 188, 189 (**Table 3**).

### sEVs application in therapy

At present, there are still quite a few diseases lacking effective treatment methods or favorable drug delivery platform, such as neurodegenerative diseases and cancers. Most of the therapeutic drugs cannot efficiently reach the lesion site across the blood-brain barrier, and the bioavailability of the drugs is relatively low. Therefore, plentiful studies have explored new therapeutic methods and found that sEVs can be used as potential non-cell therapeutics. sEVs share the advantage of low immunogenicity 190, low toxicity 191, 192, low tumorigenicity and perfect biocompatibility to cross certain biological barriers such as blood-brain barrier 129, 193. For instance, mesenchymal stem cell (MSC) sEVs are found to effectively reduce myocardial injury during myocardial infarction by transferring miR-19a, targeting SOX6, activating AKT and inhibiting JNK3/caspase-3 activation 194. Researchers found that in Alzheimer's disease, MSC sEVs derived miR-146a can be taken up by astrocytes *in vivo* and promoted the recovery of astrocyte function. Therefore, sEVs mediated miR-146a transfer may contribute to the correction of cognitive impairment in AD patients 195. Xie et al. experimentally demonstrated that miR-320a-carrying sEVs may inhibit lung cancer cell growth through the SOX4/Wnt/β-catenin axis 147. Besides, surface transmembrane proteins of sEVs enable them to target to specific recipient cells 196 by specifically binding to receptors on these recipient cells, partially endow the characteristics like tissue-specific tropism and cell-selective fusion 26. Natural tropism to liver makes sEVs ideal therapeutics for hepatic disease. Furthermore, given their natural advantages of mediating cell communication and their high physiochemical stability and biocompatibility, sEVs are considered excellent delivery platform for various therapeutic agents such as proteins, miRNAs, siRNAs, drugs, and even nanomaterials 197. Some tailored strategies are also been developed to enhance the targeting ability of sEVs to specific tissues and organs and to improve their repairing efficiency.

The potential of sEVs in disease diagnosis and treatment has also been demonstrated in clinical phase I trials. For example, Prof. Qu Jieming's team conducted a Phase 1 clinical trial on the effect of nebulized MSC-sEVs in alleviating lung injury. All volunteers tolerated nebulized MSC-sEVs with no adverse reactions within 7 days (NCT04313647) 198. Afterwards, the team also conducted a phase I clinical trial of aerosolized MSC-EVs in the treatment of acute respiratory distress syndrome and carbapenem-resistant Gram-negative bacilli pulmonary infection. In addition, clinical-grade MSC-derived exosomes (iExosomes) with KrasG12D siRNA payloads have been shown to improve pancreatic cancer mouse survival in multiple animal models 199. And launched a phase I clinical trial of iExosome's therapy for the treatment of patients with KrasG12D mutation-related pancreatic cancer (NCT03608631). When we apply sEVs to any clinical treatment, transparent reporting of vesicle manufacturing and characterization, appropriate quality control regulations, preclinical safety and efficacy data is required to ensure safety for clinical application 200 (**Table 4**).

## Conclusion

For the past few years, the popularity of sEVs is in a sustained growth while the transformation rate of strategies from bench-to-beside is not that satisfying. To improve the dilemma ahead, one of the most important things is to continue improving and developing simple, efficient, low-cost and highly reproducible isolation techniques and standardize the isolation strategy so as to ensure the accuracy of downstream experimental results and improve the comparability of research results. Overall, recent studies describing new isolation strategies for sEVs show great progress in harvesting high quality sEVs with increasingly convenient operating steps and high-level devices. The isolated sEVs that are used in preclinical studies and clinical trials show inspiring results in disease diagnosis, monitoring, and treatment. We expect that the resolution of these key issues would enable the use of sEVs as a novel strategy for clinical application with progressive isolation techniques being developed in the near future.

## Figures and Tables

**Table 1:** Comparison of sEVs isolation methods

Strategy	Principle	Time	Purity	Advantages	Disadvantages	Sample	References
Differential ultracentrifugation	According to particle density, size and shape	>4 h	Medium (with the coprecipitation and non-exosome contaminants)	Simple operation, low cost, suitable for large samples and high yield	Low repeatability, long time-consuming and destroying the integrity of sEVs	Plasma, urine, culture medium	136
Density gradient centrifugation	Mainly based on particle density	>16 h	High	Improved purity compared to UC	complex operation	Plasma, urine, culture medium	37, 49
Rate zone ultracentrifugation	Mainly based on particle size	>16 h	High	Improved purity compared to UC	The operation must strictly control the time	Plasma, urine, culture medium	46
Size exclusion chromatography	Porous stationary phase for separation by particle size	0.3 h	High	Maintain sEVs integrity, high yield and simple operation	Suitable for low upper limit of sample volume, need to be combined with other methods, high equipment cost and long time-consuming	Plasma, urine, Culture medium, cerebrospinal fluid (Universal for almost all biological fluids)	56, 60, 65, 136, 137
Precipitation	Changing the solubility and dispersibility of particles by using hydrophilic polymers	0.3-12 h	Low	High yield, simple operation, suitable for large samples	Low purity (affected by polymer)	Culture medium	63, 138, 139
Ultrafiltration	Using filtration membranes, the separation is based on particle size. Particles flow vertically to the membrane (vertical flow)	Generally <4 h	Low	Short time, simple operation, no need for equipment and additional separation reagents	Lower purity and higher rate of consumables (particles may clog the filtration membrane)	Fetal bovine serum, culture medium, Urine (10 kDa MWCO), Plasma (50 kDa MWCO)	37, 61, 140
Circulating tangential flow filtration (TFF) system	Compared to the TFF, there is an additional peristaltic pump that sends the flow to the membrane into a continuous loop.	-	High	Compared with the improved purity of UC, the isolated sEVs have higher biological activity	Adaptability to various types of biological fluids (such as plasma) is unclear	Culture medium	78
Hydrostatic filtration dialysis	Filtration-Concentration-Dialysis	-	-	Suitable for large samples. Compared with UC, the purity is improved, the sample loss is reduced, the yield is improved, and the operation is simple.	Efficiency may decrease when sample volume is greater than 200 mL	Urine, Culture medium	79, 141
Combined Phospholipid Affinity Method	Harnessing specific interactions between metal and phosphate groups on lipid bilayers	3-5 h	Medium	Less time-consuming than UC, with comparable purity	May also clog the filter membrane	urine	81
AF4	Cross flow perpendicular to the parabolic flow pattern, separated by particle size	4-5 h	High	Maintain sEVs integrity, high purity, and reproducibility	High requirements for equipment and operators, not suitable for large samples	Culture medium UF and SEC purified EVs from urine Plasma and serum (350 μm spacer, 10 kDa regenerated cellulose membrane)	38, 142
IAC-AsFlFFF system	Cross flow perpendicular to the parabolic flow pattern, separated by particle size	4-6 h	High	Highly reproducible, automated, and can process multiple samples simultaneously	Suitability for other samples is unclear	Plasma (350μm spacer, 10 kDa regenerated cellulose membrane)	72
AF4/UV-MALS	Cross flow perpendicular to the parabolic flow pattern, separated by particle size	4-6 h	High	high repeatability	Suitability for other samples is unclear	Urine	70
Immunoaffinity capture technology	Specific binding of capture molecules to sEVs surface markers	4-20 h	High	High purity to isolate specific sEVs subtypes	Low yield, high cost, disrupts sEVs biological function	Plasma, culture medium	38, 139, 143
Label-free microfluidics	Mainly chip technology designed according to sEVs physical properties (acoustic, electrical.)	-	High	Guaranteed sEVs integrity, simple operation, low cost, high repeatability, and broad application prospects	Still exploring	Plasma, culture medium	109
Synthetic peptide (Vn96) based isolation method	Specific affinity of Vn96 and HSP	-	High	High efficiency, high output, low cost, high versatility	Still exploring	Plasma, urine, culture medium and animal plasma	119
Chromatography-Based Systems	Separation based on the negative Zeta potential of the sEVs surface	-	-	Simple operation, adapts to a wide range of sample volumes, and maintains sEVs integrity	Susceptible to charged species in different biological fluids.	Culture medium	144
Magnetic bead-based ion exchange technology	ditto	-	-	ditto	ditto	Culture medium	95
Separation technology based on chitosan	ditto	-	-	ditto	ditto	Culture medium, Urine, Saliva	104
EXODUS	Introducing double-coupled harmonic oscillations into a double-film filter configuration to generate shear waves, separated primarily by particle size	-	High	Short time-consuming, relatively high yield and purity, suitable for a wide range of sample volumes, maintaining sEVs integrity, low cost, and scalability.	exploring	Plasma, urine, saliva, culture medium, tears	122
Separation technology based on chimeric nanocomposites	Physical absorption, electrostatic interactions, and biometric interactions	-	High	Higher yield and purity, better biological integrity, no need for expensive equipment	It's hard to completely distinguish it from other types of EVs.	Culture medium, urine	123
Based on SAP technology	Separation according to the water absorption properties of SAP	-	Low	Improve the sensitivity of liquid biopsies and preserve sEVs integrity	This technology is mainly concentrated, and the separation purity is low	Culture medium, urine	129
Anion exchange method	Separation based on negative surface charge of sEVs, elution at low NaCl concentration	-	High	High purity, high biological activity, high yield	unknown	Culture medium	133

**Table 2:** Possible problems (challenges) of sEVs application in clinic

Problems	Types	Current status	Improvement methods	Reference
Intrinsic problems of sEVs	Heterogeneity of sEVs	Its heterogeneity has not been standardized, and multiple properties are not discovered. Heterogeneity of sEVs in size, density, function, and origin has been reported	Keep researching	19, 148
	EV subtypes	There are currently three recognized subtypes, exosomes, MVs, and apoptotic bodies. In addition, there are discovered but not fully recognized subtypes, such as exomeres. There are also some subtypes that have not been discovered. Smaller subtypes beyond existing device thresholds cannot be identified.	Nano-flow cytometry combined with high-resolution microscopy, or the recently developed HF5, can improve detection resolution.	154, 155
Separation technology problems	Integrity of the isolated sEVs	Most of the current methods will destroy the integrity, such as UC.	Adjustment of external forces and selection of appropriate additional separation reagents can reduce integrity damage.	46, 107, 157
	Purity of sEVs	Most current separation techniques cannot avoid the co-separation of some components that overlap sEVs in physicochemical properties. It is currently impossible to balance yield and purity.	Immunocapture technology, TFF and combinatorial methods are relatively superior in terms of purity. It seems to me that the technical purity that can be isolated on the basis of sEVs-specific properties is relatively high.	38
	Reproducibility of sEVs isolation method	The current low reproducibility of separation technology is still a major challenge to limit the application.	Reduce manual work and operational complexity, such as EXODUS.	38
sEVs storage method	Selection of storage temperature and time	Most current storage methods may alter sEVs.	Try to use freshly isolated sEVs, and if you must store them, try to store them at -80°C for a short period of time.	2
Problems in the application process	The safety of clinical application	Security cannot be guaranteed.	Any therapeutic application of sEVs requires transparent reporting of data on vesicle manufacturing and characterization, appropriate quality control regulations, and preclinical safety and efficacy to ensure safety in clinical applications.	3, 148
	The rapid clearance of sEVs	Remaining to be improved.	Using biomaterials, hydrogels.	172

**Table 3:** Clinical Trials for Diagnostics

NCT	Name	Status	Diseases	Sampling	Markers	Methods
NCT05101655	Construction of microfluidic exosome chip for diagnosis of lung metastasis of osteosarcoma.	Enrolling by invitation	Osteosarcoma Pulmonary Metastases	Plasma	Exosome and its subgroups	Microfluidic chip technology
NCT05218759	Exosomes detection for the prediction of the efficacy and adverse reactions of Anlotinib in patients with advanced NSCLC.	Not yet recruiting	Non-Small Cell Lung Cancer	Blood	Exosomal miRNA	
NCT04499794	The study of exosome EML4-ALK fusion in NSCLC clinical diagnosis and dynamic monitoring.	Recruiting	Untreated Advanced NSCLC Patients, FISH Identified ALK Fusion Positive or Negative	Plasma	Exosome EML4-ALK Fusion	Exosome ALK fusion diagnosis and FISH examination
NCT05035134	Application of circulating exosomes in early diagnosis and prognosis evaluation after intracerebral hemorrhage.	Recruiting	Intracerebral Hemorrhage	Plasma	Circulating Exosomes	RNA sequencing and proteome sequencing
NCT04127591	Differential expression and analysis of peripheral plasma exosome miRNA in patients with myocardial infarction.	Unknown	Myocardial Infarction	Plasma	Exosomal miRNAs	Second-generation sequencing technology, qPCR
NCT04155359	Clinical evaluation of the miR sentinel BCa™ Test to diagnose bladder cancer in hematuria patients.	Recruiting	Bladder Cancer	Urine	Exosomal sncRNA	The miR Sentinel™ BCa test
NCT03895216	Identification and characterization of predictive factors of onset of bone metastases in cancer patients.	Recruiting	Bone Metastases	Plasma	Exosomal miRNAs and protein	Next Generation Sequencing (NGS), Triple TOF mass spectrophotometer and variation.
NCT04164966	Development of novel biomarkers for the early diagnosis of Type 1 Diabetes.	Recruiting	Type 1 Diabetes	Blood	Circulating β cell-specific exosomes	Baseline Sample Characterization
NCT03821909	Acquisition of portal venous CTCs and exosomes from patients with pancreatic cancer by EUS (CTCs).	Unknown	Pancreatic Cancer	The portal venous blood	Exosomal mRNA	RNA-seq
NCT04529915	Multicenter clinical research for early diagnosis of lung cancer using blood plasma derived exosome.	Active, not recruiting	Lung Cancer	Plasma	Exosomes	ELISA assay, Western blotting

**Table 4:** Clinical Trials for Therapy

NCT	Name	Status	Diseases	EV Type	Administration
NCT04602104	A clinical study of mesenchymal Stem cell exosomes nebulizer for the treatment of ARDS.	Recruiting	Acute Respiratory Distress Syndrome	hMSC-Exos	Aerosol inhalation
NCT04270006	Evaluation of adipose derived stem cells Exo in treatment of periodontitis (exosomes).	Unknown	Periodontitis	Adipose derived stem cells exosomes	Local injection in the periodontal pocket
NCT04389385	COVID-19 specific T cell derived exosomes (CSTC-Exo).	Active, not recruiting	Corona Virus Infection Pneumonia	COVID-19 Specific T Cell derived exosomes	Aerosol inhalation
NCT04798716	The use of exosomes for the treatment of acute respiratory distress syndrome or novel coronavirus pneumonia caused by COVID-19.	Not yet recruiting	Covid19, Novel Coronavirus Pneumonia, Acute Respiratory Distress Syndrome	MSC-exosomes	Intravenous injection
NCT04747574	Evaluation of the safety of CD24-exosomes in patients with COVID-19 infection.	Recruiting	SARS-CoV-2	CD24-exosomes	Aerosol inhalation
NCT04849429	Intra-discal injection of platelet-rich plasma (PRP) enriched with exosomes in chronic low back pain.	Recruiting	Chronic Low Back Pain, Degenerative Disc Disease.	Platelet rich plasma (PRP) with exosomes	Intra-discal Injection
NCT04276987	A pilot clinical study on inhalation of mesenchymal stem cells exosomes treating severe novel coronavirus pneumonia.	Completed	Coronavirus	MSCs-derived exosomes	Aerosol inhalation
NCT05060107	Intra-articular injection of MSC-derived exosomes in knee osteoarthritis (ExoOA-1) (ExoOA-1).	Not yet recruiting	Osteoarthritis, Knee	MSC-derived Exosomes	Intra-articular Injection
NCT03608631	iExosomes in treating participants with metastatic pancreas cancer with KrasG12D mutation.	Recruiting	Metastatic Pancreatic Adenocarcinoma, Pancreatic Ductal Adenocarcinoma	Mesenchymal Stromal Cells-derived Exosomes with KRAS G12D siRNA	Intravenous injection
